# Replicated life-history patterns and subsurface origins of the bacterial sister phyla *Nitrospirota* and *Nitrospinota*

**DOI:** 10.1038/s41396-023-01397-x

**Published:** 2023-04-03

**Authors:** Timothy D’Angelo, Jacqueline Goordial, Melody R. Lindsay, Julia McGonigle, Anne Booker, Duane Moser, Ramunas Stepanauskus, Beth N. Orcutt

**Affiliations:** 1 Bigelow Laboratory for Ocean Sciences, 60 Bigelow Drive, East Boothbay, ME 04544 USA; 2 University of Guelph, School of Environmental Sciences, 50 Stone Road East, Guelph, ON N1G 2W1 Canada; 3 Basepaws Pet Genetics, 1820 W. Carson Street, Suite 202-351, Torrance, CA 90501 USA; 4 Desert Research Institute, 755 East Flamingo Road, Las Vegas, NV 89119 USA

**Keywords:** Environmental sciences, Microbial ecology, Bacterial genetics

## Abstract

The phyla *Nitrospirota* and *Nitrospinota* have received significant research attention due to their unique nitrogen metabolisms important to biogeochemical and industrial processes. These phyla are common inhabitants of marine and terrestrial subsurface environments and contain members capable of diverse physiologies in addition to nitrite oxidation and complete ammonia oxidation. Here, we use phylogenomics and gene-based analysis with ancestral state reconstruction and gene-tree–species-tree reconciliation methods to investigate the life histories of these two phyla. We find that basal clades of both phyla primarily inhabit marine and terrestrial subsurface environments. The genomes of basal clades in both phyla appear smaller and more densely coded than the later-branching clades. The extant basal clades of both phyla share many traits inferred to be present in their respective common ancestors, including hydrogen, one-carbon, and sulfur-based metabolisms. Later-branching groups, namely the more frequently studied classes *Nitrospiria* and *Nitrospinia*, are both characterized by genome expansions driven by either de novo origination or laterally transferred genes that encode functions expanding their metabolic repertoire. These expansions include gene clusters that perform the unique nitrogen metabolisms that both phyla are most well known for. Our analyses support replicated evolutionary histories of these two bacterial phyla, with modern subsurface environments representing a genomic repository for the coding potential of ancestral metabolic traits.

## Introduction

Approximately 13% of Earth’s biomass—and 80% of all bacterial and archaeal biomass—is estimated to be located within the subsurface [1, 2]. Recent advances in field technologies have allowed for expansive sampling of this biomass in the terrestrial and marine subsurface [3]. Many of these subsurface environments have been suggested as possible locations for the origins of life or to retain signatures of early evolutionary history [4–9]. Analysis of nucleic acids from subsurface biomass has allowed for a broader understanding of the characteristics of microorganisms that inhabit these habitats [10–14]. Several groups of primarily subsurface-inhabiting *Bacteria* and *Archaea* are presumed to have retained ancient traits due to the environments being analogous to early-Earth, in some cases isolated from the surface world on geologic timescales [15, 16].

The phyla *Nitrospirota* and *Nitrospinota* both share nitrite-oxidizing metabolisms and have long been considered to be sister phyla [17–19]. Confirming this evolutionary relationship, recent systematic reconstructions of the tree of life have placed these two phyla as direct relatives [20–23]. Both phyla have cosmopolitan distributions and are present in a large variety of environments, including deep terrestrial and marine subsurface environments. For example, the more well-studied groups of the *Nitrospirota* (class *Nitrospiria*) and *Nitrospinota* (class *Nitrospinia*) are commonly detected in marine environments, activated sludge, soil, drinking-water, and waste-water treatment plants [24–33]. These taxa are known for their nitrite oxidization and complete ammonia oxidation “comammox” metabolisms [24–33]. By contrast, the *Thermodesulfovibrionia* class of *Nitrospirota* is not common in surface environments but have frequently been sampled from in marine and terrestrial subsurface aquifers [11, 12, 34–38]. Members of the *Thermodesulfovibrionia* class have different physiologies than the *Nitrospiria* class, including hydrogen oxidation, sulfate reduction, nitrate reduction and sulfur disproportionation [36–40].

Though the shared trait of nitrite oxidation has long been known, a broader comparison of these sister phyla has not yet been performed. Here we explore and compare the functional characteristics of these phyla along their evolutionary histories in order to fill that knowledge-gap. We use phylogenomic, functional, and gene-tree-based methods to establish the connection of basal clades subsurface environments and reveal patterns of metabolic expansion driven by a combination of vertical evolution and horizontal gene transfer. These analyses document a partially replicated evolutionary history of these sister phyla which demonstrates how multiple modes of evolution can shape closely related phyla that occupy similar niches.

## Materials and methods

### Genomic dataset collection, curation, and quality control

This study used publicly available genome assemblies as well as newly generated datasets (Supplemental Methods). Existing publicly available genome assemblies were downloaded from the National Center for Biotechnology Investigation (NCBI) and the Integrated Microbial Genomes (IMG) database of the U.S. Department of Energy’s Joint Genome Institute in June 2021. The Genome Taxonomy Database (GTDB) website (release 202) [41] was used to access lists of NCBI assembly accession numbers for the following GTDB-assigned phyla: *Nitrospinota*, *Nitrospinota_A* (now called *Tectomicrobia*), *Nitrospinota_B*, *Nitrospirota*, *Nitrospirota_A* (*Leptospirilla*). The IMG assemblies were found using the same GTDB taxonomy classifier using the search function on the IMG website. IMG metagenome assemblies that were designated as “public” and “published” were also downloaded for these phyla. Duplicate entries between IMG and NCBI were manually removed.

New single-cell amplified genomes (SAGs) from several field sites that were recently made public were used. These include subsurface hydrothermal fluids in September 2018 from the marine serpentinizing Lost City hydrothermal vent field (NCBI BioProject PRJNA779602, Supplemental Table 1), collected in April 2021 from a continental fracture fluids of the Death Valley Regional Flow System (Amaragosa Valley, USA), via the Inyo-BLM1 well (NCBI BioProject PRJNA853307, Supplemental Table 2), and deeper sequenced SAGs collected in 2015 from the of the Atlantis Massif that hosts Lost City, originally described in reference [13] (NCBI BioProject PRJNA825747, Supplemental Table 3). Detailed information on the generation of these new SAGs is available in the Supplemental Methods file.

Quality control of the assemblies was performed using the CheckM qa workflow (v 1.07) to remove genomes with <50% genome completion and >10% sequence contamination, leaving genomes that fall within the MIMAG categories “medium” (>50% completion, <10% contamination) and “high” (>90% completion, <5% contamination) [42, 43]. These resulting genomes were dereplicated with dRep, using default parameters, to remove nearly-identical assemblies [44]. All genomes were then classified using the GTDB-tk classifier tool (v1.5.0, r202) [41] (Supplemental File 1). In the methodology described below, polyphyletic groups that were once considered a part of *Nitrospirota* and *Nitrospinota* (i.e., *Nitrospirota_A* (*Leptospirilla*), *Nitrospinota_A* (*Tectomicrobia*), and *Nitrospinota_B*) were included only in the phylogenomic trees. These groups were not included in the gene cluster based functional analyses. All code to recreate these processes are available at https://github.com/ts-dangelo/bioinformatic_scripts_python and outlined in Supplemental Fig. 1.

### Phylogenomic analysis

Phylogenies of the individual phyla were constructed using the PhyloPhlan pipeline with the Bac120 conserved marker-protein database R202) [41, 45]. The PFAM and TIGRFAM protein files for the Bac120 database contain 218–248 k sequences per file and are too large for the memory requirements of Diamond [46]. Therefore, each protein family in the Bac120 database was randomly subset to 1000 sequences per family. For comparison, the default PhyloPhlan marker protein database contains 337–1344 sequences per protein family. The subsampled version of the Bac120 database was used to create a custom PhyloPhlan database using the command phylophlan_setup_database. The default PhyloPhlan pipeline was run with the -min_num_markers flag set to 12, using the default parameters for the *--diversity high* and *–accurate* settings (additional details in Supplemental Info) [45]. The alignments of individual marker genes were concatenated into one file and used as input for IQ-TREE (v2.0.3) using the parameters -m TEST -bb 1000, where ModelFinder was used to choose the most appropriate model by the Bayesian Information Criteria (BIC) [47, 48]. *Desulfobacterota_D* (*Dadabacteria*) and class *Thermodesulfobacteria* (Phylum *Desulfobacterota*) were used as outgroups. Polyphyletic phyla (*Nitrospirota_A*; *Leptospirilla*, *Nitrospinota_A* (*Tectomicrobia*), and *Nitrospinota_B*) were included in their respective phylogenies. Relative Evolutionary Divergence (RED) scaling was used to display appearance of certain metabolic traits in relative time along the phylogeny of the investigated phyla [41].

### Gene clustering, gene tree production, and gene reconciliation

Open reading frames were identified in genome assemblies by Prodigal (v2.6.3), using the default parameters of anvi-gen-contigs-database in the Anvi’o analysis pipeline (v7) [49, 50]. The amino acid sequences for all assemblies were clustered into gene clusters using Diamond and the Markov Cluster Algorithm (MCL) with an inflation parameter of 1.2, after blast-hits were filtered using the MINBIT parameter of 0.5 [46, 51, 52]. The resulting gene clusters were exported from Anvi’o and assembly × gene cluster count matrices for each phylum were created from the data using a custom Python script. Matrices were pruned to only contain gene clusters present in at least four genomes and then converted to presence/absence. These matrices were used to hierarchically cluster genomes by gene content using Ward’s linkage method. Gene clusters were annotated with the eggNOG database (version 5.0) using the eggNOG emapper (version 2) using default parameters [53]. In addition, KOFAMSCAN was used to annotate gene clusters. The default thresholds of the “exec_annotation -f mapper” command were used [54]. Consensus annotation for each gene cluster was created by tallying the annotations assigned by eggNOG and KOFAMSCAN for each sequence in a given gene cluster and choosing the most frequent annotation as the consensus annotation, respectively (Supplemental Files 2–7).

Gene trees were constructed for each gene cluster by aligning the gene cluster amino-acid file with MAFFT (v7.490, options –retree 2), trimming the alignments with TRIMAL (v1.2, -automated1 -resoverlap 0.55 -seqoverlap 0.6) and constructing trees with IQ-TREE (v2.0.3, using ModelFinder to identify the most appropriate model via BIC and 1000 UltraFast non-parametric bootstraps (UFboot)) [47, 48, 55, 56], similar to other recent analyses [57, 58]. To calculate the location of the gene originations of enriched gene clusters (described below), gene trees were reconciled against the phylogenomic tree (species tree) using the standard workflow of GeneRax [59]. Gene trees were constructed per phylum, as described above, and were reconciled to the phylogenomic trees of the individual phylum (the phylogenetic relationships of gene clusters of particular interest were investigated in detail, described below). The phylogenomic trees used for reconciliation methods were rooted using the Minimum Ancestor Deviation method (MAD) [60]. This was done to circumvent dataset size and complications of including an outgroup for this data analysis. Testing showed that trees rooted with MAD have nearly identical topologies as outgroup rooted trees, with minor differences only occurring at nodes that were not bootstrap supported (UFboot >95%, Supplemental Figs. 2, 3).

Broader relationships of gene cluster of interest (*nxrA*, *dsrA*) were investigated. All GenBank amino acid sequences annotating to *nxrA* nitrite oxidoreductase were downloaded and the gene cluster sequences were aligned with the GenBank sequences using MAFFT (--auto) and the alignments were trimmed using trimAL (-automated1) [55, 56]. A phylogeny for *dsrA* was made using the RefSeq-quality Bacterial sequences in TIGRFAM02064. Phylogenies were constructed using IQ-TREE as described above. The *nxrA* tree was rooted with tetrathionate reductase (*ttrA*) from *Desulfobacterota* and the *dsrA* was rooted on the *Firmicutes* clade, as done elsewhere [61].

### Statistical analyses

Gene clusters that were differentially distributed in the major delineations identified by hierarchical clustering (mainly corresponding to the taxonomic class) were identified using the proportional generalized linear model incorporated into the Anvi’o package [50]. Enriched gene clusters were filtered by the heuristics of: (1) being present in a given clade more than expected by chance, (2) being above a significant *q* value threshold, and (3) further filtered to the gene clusters occurring in less than 10% of the genomes of the other clade besides the focal clade. These heuristics were used to focus on genes that are highly prevalent in a given clade. To determine statistical differences between genome properties (estimated length and coding density) analysis of variance (ANOVA) was performed using the f_oneway command in the SciPy Python library [62].

Ancestral state reconstruction (ASR) facilitated by MrBayes (v3.2.7a) was used to reconstruct the approximate traits of the respective ancestors of the phyla *Nitrospirota* and *Nitrospinota*. The gene clusters in the given phyla were prevalence filtered to only include clusters present in >10% of the genomes. The presence or absence of a gene cluster was treated as a binary state variable and the MAD-rooted phylogeny of the phyla was used to estimate the probability of the state of a given gene cluster at the internal nodes of the tree using the MrBayes Markov Chain Monte Carlo (MCMC) sampler with 500,000 MCMC samples [63, 64]. Gene clusters having a greater than 0.5 posterior probability (pp) at the root node were interpreted as potentially present in the ancestral relative of the given phyla. These ancestral state reconstruction results were compared to the gene-tree species-tree reconciliation results, with the assumption that gene clusters likely present at the root of the phylogenomic tree would have their origination event deep within the tree, at or close to the root node. The habitat-type where genomes were sampled were coded as variables to infer the probability that the ancestral node of the given phyla occupied a particular environment type using MrBayes (v3.2.7a). The NCBI BioSamples that produced the given assemblies were aggregated in sample-type groups using the “isolation source” metadata on the NCBI website. The classifications are outlined in columns “P” and “Q” of Supplemental Data 1.csv.

## Results

### Phylogenetic and gene content clustering patterns

**Fig. 1:**
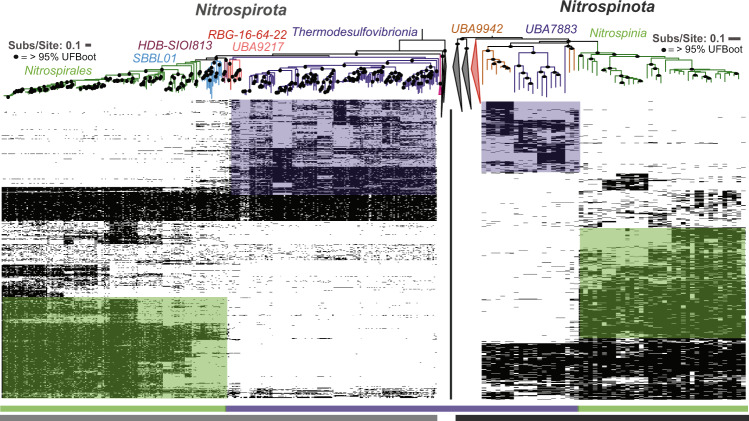
Phylogenomic trees and gene content of *Nitrospirota* and *Nitrospinota* phyla, showing distinct clades and gene clusters associated with basal groups comprised mainly of subsurface organisms (purple bar at bottom) or later-branching groups (green bars at bottom). The trees are oriented so the middle of the image contains the outgroups for both trees. Phylogenetic trees were produced used the Bac120 marker set (min 12 genes) using the PhyloPhlan pipeline to create the alignments and IQ-TREE using the LG + F + G4 model chosen by ModelFinder using the Bayesian Information Criterion (BIC) and 1000 ultra-fast non-parametric bootstraps. Heatmaps of gene cluster absence (empty) or present in at least 10% of the genomes in the given phyla (filled) are displayed below the trees. Rows of gene clusters are ordered by hierarchical clustering using wards linkage. Blocks of genes enriched in the *Nitrospinia* or *Nitrospiria* are highlighted with the green boxes, and purple used to highlight the enriched gene clusters in the basal classes of the given phyla. Hierarchical clustering of genomes solely by gene content produced similar groupings as the phylogenies (Supplementary Figure 5).

**Fig. 2:**
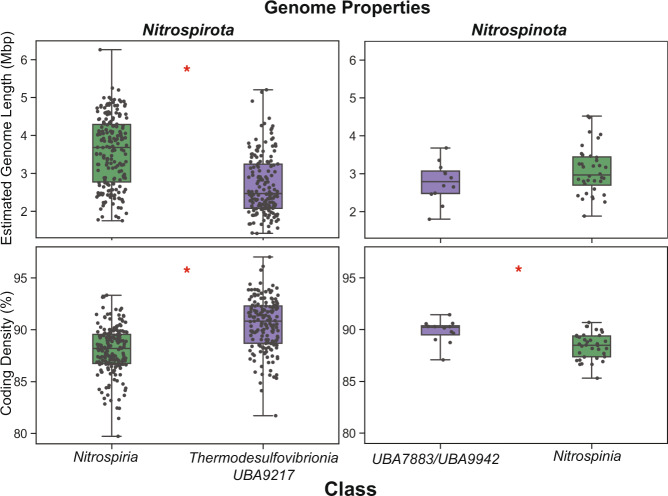
Genome properties via CheckM of the major phylogenetic delineations (also corresponding to gene cluster content) in *Nitrospirota* and *Nitrospinota* show that basal clades (purple) have smaller genome length and higher coding density than later brancing clades (green). Estimated genome length y-axis scale is 10^6^ base pairs, and coding density y-axis is as percent. Red asterics denote results that show significant differences between metrics in the clades by a ANOVA (*p* value < 0.05). For *Nitrospirota* the *p* values for genome length and coding density are 3.63e^−20^ and 3.25e^−20^, respectively. For *Nitrospinota* the *p* values for genome length and coding denisty are 0.13 and 0.0011, respectively.

The quality-controlled public data and newly released SAG data contained 367 and 57 assemblies belonging to the phyla *Nitrospirota* and *Nitrospinota*, respectively (Supplemental Fig. 4). Phylogenomic analysis of *Nitrospirota* shows that *Thermodesulfovibrio*, *RBG-16-64-22*, and *UBA9217* are earlier branching, basal classes while *Nitrospiria* is a later-branching class within the *Nitrospirota* phylogeny (Fig. 1, Supplemental Figs. 2, 3). Likewise, phylogenomic analysis of *Nitrospinota* shows classes *UBA7883* and *UBA9942* as earlier branching basal groups, with *Nitrospinia* as a later branching class. The earlier branching classes in both of these phyla tend to have smaller genomes with higher coding density (% of total base pairs contained within open reading frames) (ANOVA *P* < 0.05 except for *Nitrospinota*, where early-branching genome lengths had a smaller mean, but a non-significant *p* value; Fig. 2).

**Fig. 3:**
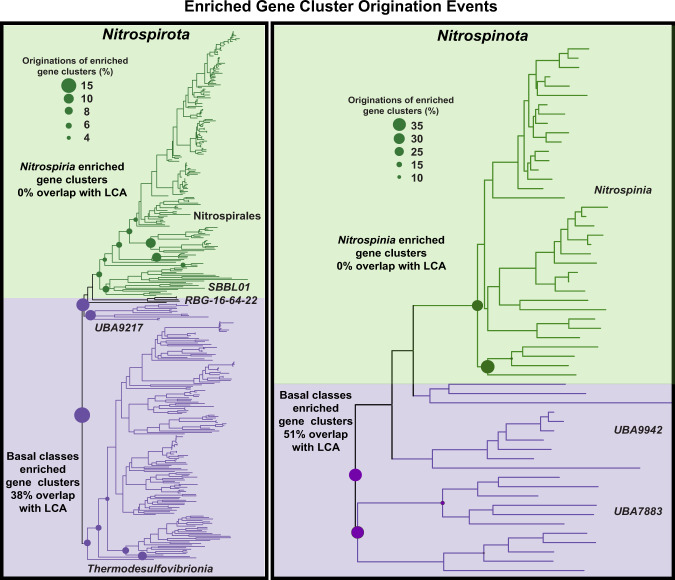
Gene origination events of *Nitrospirota* and *Nitrospinota* for gene clusters enriched in basal and later-branching clades (as shown in Fig. 1). The size of the circles represents the percentage of the enriched gene clusters subset (basal enriched or later-branching enriched) that had its initial origination event at the given node. Gene clusters enriched in basal clades of both phyla show overlap with gene clusters of the Last Common Ancestor (LCA) identified by Ancestral State Reconstruction and have their originations at the root or in basal nodes close to the root (purple, both sides). Gene clusters enriched in later-branching clades do not overlap with LCA gene clusters and have most of their originations at the base of classes *Nitrospiria* and *Nitrospinia*, respectively (green, both sides).

Hierarchical clustering of these phyla by gene content also shows distinct separation of the class *Nitrospiria* from the classes *Thermodesulfovibrio*, *RBG-16-64-22*, and *UBA9217* of the *Nitrospirota* (Fig. 1, Supplemental Fig. 5). Similar gene content clustering is apparent with class *Nitrospinia* distinct from basal classes *UBA7883*/*UBA9942* in *Nitrospinota*. The later-branching classes of both phyla contain more enriched gene clusters than the basal classes. In the *Nitrospirota* there are 1127 gene clusters enriched in the *Nitrospiria* and 486 gene clusters enriched in *Thermodesulfovibrio*/*UBA9217*/*RBG-16-64-22*. In *Nitrospinota* there are 871 gene clusters enriched in the *Nitrospinia* and 412 gene clusters enriched in classes *UBA7883* and *UBA9942* (Fig. 1, Supplemental Fig. 5).

Classification of the sampling sites that produced these assemblies using NCBI metadata shows that most members of the earlier branching classes in both *Nitrospirota* and *Nitrospinota* (i.e., *Thermodesulfovibrio*, *RBG-16-64-22*, *UBA9217*, *UBA7883*, *UBA9942*) were sampled from marine or terrestrial subsurface aquifers. The later-branching classes of *Nitrospirota* contain a mixture of subsurface and surface inhabitants while later-branching *Nitrospinota* (*Nitrospinia*) are comprised mainly of assemblies sampled from marine environments, including deep water layers (Supplemental File 1, Supplemental Figs. 6, 7, 8). Ancestral state reconstruction (ASR) using broad environmental categorization suggests that the ancestral nodes of both *Nitrospirota* and *Nitrospinota* have high posterior probability of resembling assemblies sampled from terrestrial subsurface aquifers (99% pp, 87% pp, respectively; Supplementary File 1, Supplemental Figs. 6, 7, 8).

### Patterns of enriched gene clusters

In the *Nitrospirota* phylum, of the 4598 gene clusters present in >10% of the assemblies, there were 823 gene clusters identified by ancestral state reconstruction (ASR) to have >0.5 pp at the root node. Gene-tree reconciliation methods show that the majority of these gene clusters (84%) had their originations between the root and the three deepest nodes of the tree (Fig. 3), indicating good agreement between methods. Of these gene clusters identified by ASR, 187 also belonged to the 485 gene clusters identified as enriched in the early branching basal classes *Thermodesulfovibrio*/*RBG-16-64-22*/*UBA9217* by the proportional GLM test (38%). These gene clusters have their origination events at basal nodes in the phylogeny (Fig. 3). None of the 1127 gene clusters enriched in the later branching *Nitrospiria* class were identified by ASR to have >0.5 pp at the root node. Gene-tree species-tree reconciliation shows that the gene clusters enriched in *Nitrospiria* do not originate until early nodes in the class *Nitrospiria* and order *Nitrospirales* (Fig. 3).

In the *Nitrospinota*, of the 3901 gene clusters present in >10% of the assemblies, there were 761 gene clusters with >0.5 pp at the root node identified by ASR, and 88% of these gene clusters had their origination event at the root or between the root and the three deepest nodes of the tree (Fig. 3). Of these gene clusters, 212 also belonged to the 412 gene clusters enriched in the basal Nitrospinota classes (51.4%). Reconciliation methods show these gene clusters originated near the base of the phylogeny (Fig. 3). In contrast, none of the ancestral gene clusters overlapped with significantly enriched gene clusters in the class *Nitrospinia*, the same pattern seen in *Nitrospirota*. Reconciliation methods indicate that the majority of these gene clusters have their original speciation event at one of the four deepest nodes in the class *Nitrospinia* (Fig. 3).

### Energy production and conversion traits inferred from LCA gene clusters

**Fig. 4:**
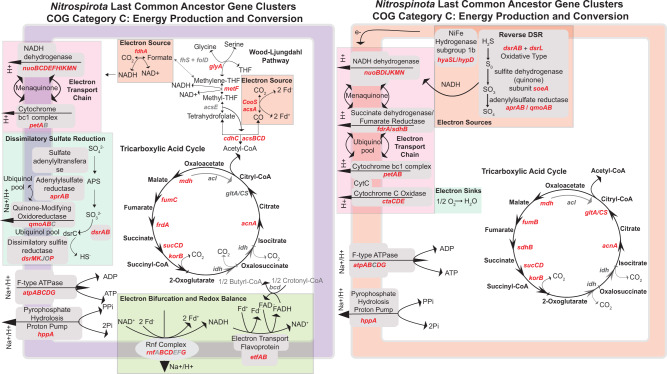
Genome diagrams of COG Category C: Energy Production and Conservation gene clusters in putative last common ancestors (LCA) of *Nitrospirota* and *Nitrospinota* identified by ASR (0.5 > posterior probability) at the root node of the respective phylogenies. Gene symbols in red are present, gene symbols in gray are genes that are a part of the given pathway depicted, but are not present or are not above the 0.5 > pp threshold for inclusion in the LCA gene cluster set.

The putative traits of the last common ancestor (LCA) of the *Nitrospirota* depict an organism that uses one-carbon compounds as electron sources (Fig. 4). Genes for formate oxidation (*fdhA*) and carbon monoxide oxidation (*cooS*/*acsA*) are present as the only genes indicative of an electron source in the dataset. The carbon monoxide dehydrogenase complex *cooS*/*acsA* exists as part of the Carbonyl branch of the Wood-Ljungdahl Pathway (WLP) with other components of the methyl-transferase module of the WLP (*acsCD*) [65]. The *rnf* complex is present, which allows for the production of NADH and a proton gradient from reduced ferredoxin produced from CO oxidation [65, 66]. The electron bifurcating complex *etfAB* is also present, which participates in FAD and ferredoxin recycling [67, 68]. All components of the NADH dehydrogenase *nuo* operon and one subunit of the cytochrome bc1 complex are present for proton-motive force production. The majority of the dissimilatory sulfate reduction pathway is observed (*qmoAB*/*AprAB*/*dsrABMKOP*). Additional sources of proton-motive force may be produced by the pyrophosphate-hydrolysis powered proton pump *hppA*. Energy conservation is performed via an F-type ATPase. Gene clusters annotating as the methyl branch of the WLP that contains the enzymes to integrate Acetyl-CoA metabolism into Glycine/Serine biosynthesis are present in the LCA dataset [69]. The oxygen detoxification genes super-oxide dismutase *sodA* and rubredoxin are present in the LCA gene clusters (Fig. 4).

The traits of the LCA of *Nitrospinota* depict an ancestor that uses sulfur compounds and hydrogen as electron sources (Fig. 4). The genes for subunits of subgroup 1b NiFe hydrogenase are present (*hyaABCD*). The same gene clusters for *dsrAB* present in the LCA of *Nitrospirota* are present in the LCA of *Nitrospinota*. A phylogenetic analysis of this *dsrA* gene cluster with RefSeq representatives of TIGRFAM02064 (*dsrA*) shows *Nitrospinota* and *Nitrospirota RBG-16-64-22 dsrA* sequences forming a clade close to sequences from sulfide oxidizing *Alphaproteobacteria* [61, 70] (Supplementary Figure 9). The sulfide oxidation via reverse dissimilatory sulfate reduction (rDSR) accessory protein *dsrL* is present in the LCA gene clusters [71]. In addition, present are sulfur-metabolism gene clusters annotated as the membrane-bound sulfite dehydrogenase (*soeA*/*dmsA*) responsible for sulfite oxidation to sulfate with oxygen [72, 73]. A proton-motive force is generated by NADH dehydrogenase and an electron transport chain (Fig. 4). Oxygen is used as a terminal electron acceptor via cytochrome c oxidase (*ctaCDE*). Similar to the *Nitrospirota* LCA dataset, the same F-type ATPase is present along with *hppA*. Core carbon anabolism/catabolism is performed by the TCA cycle, which has been described in representatives of this phylum [17].

### Common and contrasting metabolic properties of early and late branching clades

**Fig. 5:**
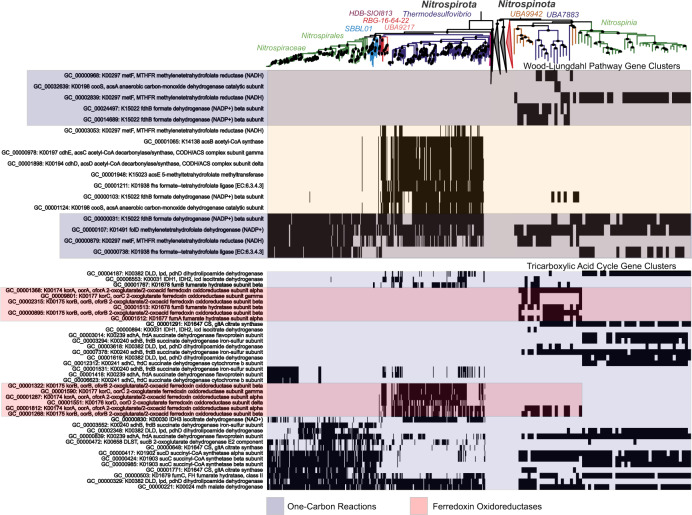
Presence/absence patterns of gene clusters with annotations involved in the Wood-Ljungdahl Pathway (WLP, KO Ids included in KEGG Module M00377) and tricarboxylic acid cycle (TCA, KO Ids included in KEGG Modules M00009-11) in *Nitrospirota* and *Nitrospinota*. The rows are ordered by hierarchical clustering of presence/absence patterns in *Nitrospirota* using Ward’s Linkage. Functional differences and similarities between the two phyla can be noted, namely the use of WLP in early-branching *Nitrospirota* and the shared use of the rTCA cycle in *Nitrospirales* and *Nitrospinota*. The purpled shaded boxes denote gene clusters with annotations involving C1 metabolisms, the red shaded boxes denote *kor*/*oor* annotated gene clusters likely involved in low redox-potential ferredoxin cycling present in basal groups of both phyla.

In both phyla, early branching clades have metabolic capabilities that are absent in later branching clades (Figs. 3, 5). Gene clusters with annotations as 2-oxoacid oxidoreductases (*korABC*/*oorABC*), involved in the rTCA cycle and also playing roles in ferredoxin cycling, one and two-carbon compound metabolisms, and low-potential electron transfers are only present in the basal clades of *Nitrospirota* and *Nitrospinota* (Fig. 5) [74]. Gene clusters involved in the WLP are present in the class *Thermodesulfovibrio* and absent in class *Nitrospiria* of *Nitrospirota* and completely absent in *Nitrospinota* (Fig. 5). Citrate-synthase (*CS*/*gltA*) and the other components of the TCA/rTCA cycle are present in the class *Nitrospiria* of the *Nitrospirota* and present throughout the *Nitrospinota* (Fig. 5) [75, 76].

**Fig. 6:**
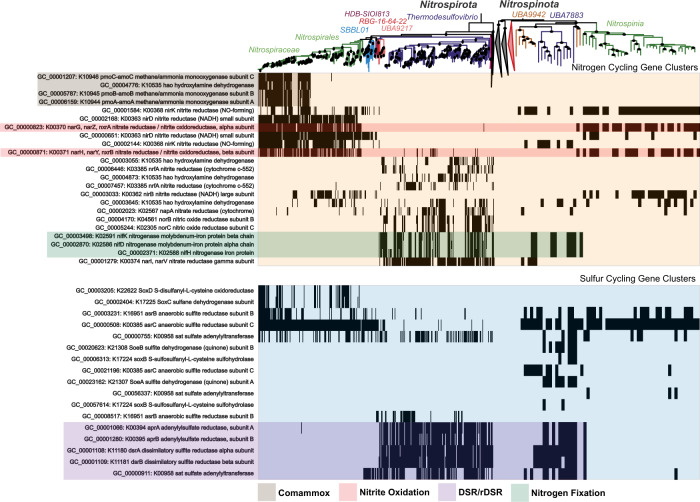
Presence/absence patterns of gene clusters with annotations involved in the Nitrogen cycling (KO Ids included in KEGG Map M00910) and Sulfur cycling (KO Ids included in KEGG Map M00920) in *Nitrospirota* and *Nitrospinota*. The rows are ordered by hierarchical clustering of presence/absence patterns in *Nitrospirota* using Ward’s Linkage. Functional differences and similarities between the two phyla can be noted. The shaded boxes denote functions of interest that are discussed in the text.

In both phyla, gene clusters involved in nitrogen fixing *nif* operon are enriched in the early-branching classes, but absent in the later branching classes (Fig. 6). Although they did not pass the thresholds used for inclusion into the putative LCA gene cluster dataset, many gene clusters involved in dissimilatory nitrate reduction processes are present in *Thermodesulfovibrio* genomes (Fig. 6) [39, 40]. Terminal oxidases are also sporadically present in *Thermodesulfovibrio* (Supplementary Figure 10). Unique cytochromes and other genes known to be involved in manganese oxidation are present solely in order *SBBL01* (also referred *Ca. Manganitrophaceae*), which have been analyzed in detail recently (Fig. 8, Supplementary Figure 10) [77, 78].

**Fig. 7:**
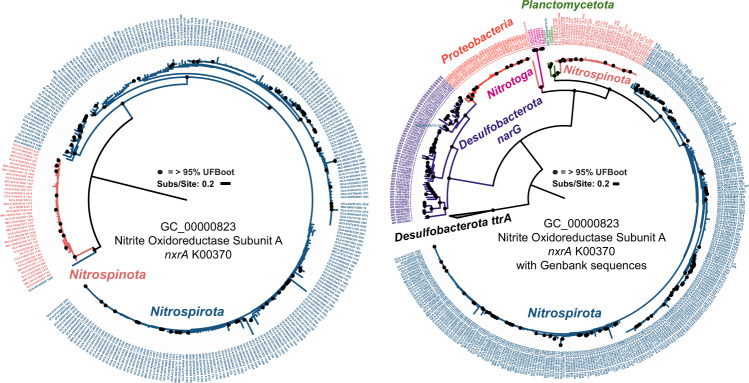
Phylogeny of gene cluster GC_00000823, annotated as K00373 Nitrite Oxidoreductase Subunit A. **A** Displays the phylogeny of the gene cluster rooted at the branch separating the majority of the *Nitrospirota* sequences from the *Nitrospinota* sequences. The LG + G4 model was used, as chosen by the B.I.C by ModelFinder. A highly divergent sequence from GCA_016212295 (*Nitrospinota*) was removed from the left panel for ease of visualization. This sequence can be seen as a diverged relative of Nitrotoga *nxr*A sequences on the right panel. **B** Displays gene cluster GC_00000823 sequences with all *nxr*A from GenBank. Respiratory *narG* from *Desulfobacterota* was included as well as tetrathionate reductase A (*ttrA*) from *Desulfobacterota*, that was used as an outgroup. The LG + I + G4 model was used, as chosen by the B.I.C by ModelFinder. The sequence from *Nitrospirota* assembly GCA_003354025, which branches closer to the *Nitrospinota* in the left panel is within the *Planctomycetota* on the right panel.

**Fig. 8:**
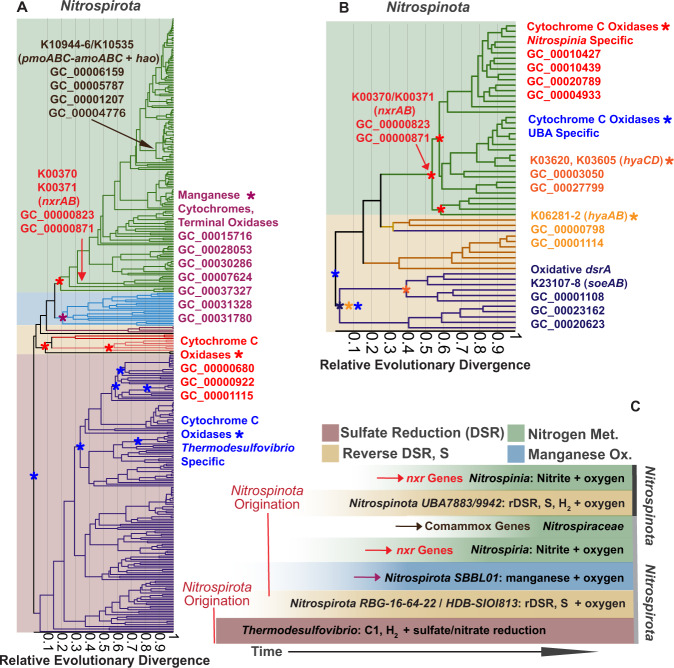
Proposed scheme of metabolic progression throughout the histories of Nitrospirota and Nitrospinota. **A** The phylogeny of *Nitrospirota* scaled with Relative Evolutionary Divergence (RED) (x axis), as used to denote particular taxonomic ranks by GTDB. The branches are colored in the same fashion as Fig. 1. The taxonomic groups are colored by boxes denoting the main metabolisms of those groups as defined in panel (**C**). **B** RED scaled phylogeny of *Nitrospinota*. Gene cluster originations, as determined by gene reconciliation with Generax, of gene clusters of interest are denoted by colored asterisks and arrows. **C** Schematic interpretation of the data in panels (**A**) and (**B**). Sulfate respiring *Nitrospirota* originate first. The second-branching groups of *Nitrospirota* perform reverse dissimilatory sulfate reduction (rDSR), while *Nitrospinota* using this metabolism likely arises around the same time. After rDSR in *Nitrospirota*, groups performing manganese oxidation form. The last acquired metabolisms in both phyla are the nitrogen-based metabolisms involving nitrite oxidation genes and comammox genes.

Gene clusters involved in nitrite-based metabolisms and comammox metabolisms are absent in basal lineages of both phyla but are present in both later branching *Nitrospiria* and *Nitrospinia* classes (Fig. 6). Both phyla have the same enriched gene clusters for nitrite oxidoreductase (*nxrAB*) (Fig. 7). The phylogeny of the *nxrA* gene cluster is largely monophyletic (Fig. 7A). Additional phylogenetic analysis of this gene cluster with *nxrA* sequences from GenBank suggest that these sequences from both phyla have transfer histories with the *Planctomycetota* (Fig. 7B) [19]. Gene-tree reconciliation methods indicate acquisition of these genes by the phyla early within the later branching *Nitrospiria*/*Nitrospinia* classes (Fig. 8). Gene clusters with annotations ammonia monooxygenase and hydroxylamine dehydrogenase (*pmo-amoABC*, *hoa*), involved in comammox metabolism, originate at the base of the *Nitrospiraceae* family (Figs. 6, 8).

## Discussion

### Shared evolutionary traits of *Nitrospirota* and *Nitrospinota*

Recent systematic reconstructions of Bacterial phylogeny and evolution place the *Nitrospirota* and *Nitrospinota* as direct relatives [20–23]. This relationship has long been considered due to their shared nitrite-oxidizing metabolisms and the observation that orthologous proteins from *Nitrospina gracilis* of the *Nitrospinota* have *Nitrospira* of the *Nitrospirota* as a most frequent neighbor [17]. Our analysis demonstrates that these two sister phyla share several traits throughout their histories besides nitrite oxidation. These include the primarily subsurface-inhabitation of basal clades that use sulfur-based metabolisms, followed by expanded metabolic capabilities in later branching clades. These changes appear driven by genome expansion and a combination of gene gain and loss. The phylum *Nitrospirota* contains a more diverse set of metabolic capabilities than *Nitrospinota* and the metabolic capabilities of the LCA gene clusters of the two phyla suggest that Nitrospirota is older (i.e., Formate, CO oxidization, WLP) [7, 8].

Gene clusters identified as enriched in the basal clades overlap with gene clusters likely to be present in the LCA (Figs. 3, 4). These results suggest that the extant subsurface-inhabiting members share many traits with the ancestors of these phyla. In contrast to the basal clades, gene clusters identified as enriched in the later-branching clades show no overlap with the LCA gene clusters. Gene-tree reconciliation techniques show that these gene clusters originated at the base of the later-branching classes (Fig. 3). The genomes of these later branching classes are larger (Fig. 2), suggesting these phyla have undergone genome expansion. This is exemplified by the acquisition of notable nitrogen-cycling genes from other phyla (Figs. 7, 8). It is interesting to note that *Dadabacteria* (a relative to *Nitrospirota* and *Nitrospinota* [20, 21]) presents an opposing pattern, where early branching clades contain many genomes from subsurface organisms that have larger genomes than later-branching marine clades [79]. This suggests that genome expansion and streamlining patterns are influenced by the particular metabolisms and niche occupation of a given group of organisms. This has been noted in *Thaumarchaeota* and *Cyanobacteria*, where lateral gene transfer and duplication are associated with occupation of terrestrial niches while gene loss is more prevalent in clades that live in marine environments [57, 80, 81].

### Ancestral metabolisms inferred from LCA gene clusters

Ancestral state reconstruction of the traits with a >0.5 pp at the root of *Nitrospirota* depict a physiology very different from the more well-studied order *Nitrospirales* [18, 19, 26, 28–30, 32, 33]. The gene cluster annotations suggest a C1-compound-based metabolism that utilizes formate and CO and reduces sulfate as an electron acceptor (Fig. 4). Formate and CO are used as electron sources for deeply-branching bacteria and archaea [15, 16] and they can be produced abiotically in the hydrothermal environments were many of these genomes were sampled from [4, 5]. A representative of the class *Thermodesulfovibrio* which was isolated from the terrestrial subsurface can perform sulfate reduction with hydrogen [35]. Although hydrogen oxidation genes were not present in the LCA dataset based above the >0.5 pp threshold used, genes with these annotations are present in the subsurface-enriched subset of gene clusters (Supplemental Data File 2). The same is true of several gene clusters involved with dissimilatory nitrate reduction, which has been documented in members of the *Thermodesulfovibrio* (Fig. 6) [39, 40].

A phylogeny of the gene cluster that annotates as *dsrA* with sequences from TIGR02064 has a similar topology to a study concluding that some *Nitrospirota* and *Nitrospinota* use *dsr* genes in reverse to oxidize hydrogen sulfide [61]. The presumed oxidative *dsrA* sequences from *Nitrospirota* and *Nitrospinota* form a clade, which contains sequences from *Ca. Magnetaquicoccus inordinatus*, that is basal to most sulfide oxidizing *Alphaproteobacteria* [70]. This topology suggests *Nitrospirota*/*Nitrospinota* oxidative *dsrA* sequences share a history with sequences belonging to a sulfide-oxidizing Proteobacterial ancestor (Supplementary Figure 9). A gene cluster annotating as *dsrL*, which acts as an accessory protein involved in rDSR in *Allochomatium vinosu* is present in the *Nitrospinota* LCA and but only 7/367 (0.27%) of *Nitrospirota* genomes [71]. Four of these *Nitrospirota dsrL* sequences belong to genomes in the clade containing class *RBG-16-64-22* and one from an assembly from the early-branching *Nitrospiria* order *HDB-SIOI813*. The assemblies in order *HDB-SIOI813* contain incomplete *dsr* operons and other sulfur-metabolism-related genes such as *aprAB* and *sat* (Fig. 6). These gene content patterns suggest these groups perform rDSR or metabolize other sulfur-cycle intermediates, which has been observed in other species with these genes, such as *Desulfurivibrio alkaliphilus* and some *Acidobacteria* [82–84] (Figs. 6, 8).

Time-calibrated phylogenies of Bacterial evolution suggest the basal node of the close relatives of *Nitrospirota*—*Acidobacteria* and *Desulfobacterota*—originated just prior to, or around the time of the great oxidation event (GOE) [22, 23, 85–87]. The time of origin of these phyla closely coincides with a proliferation of oxygen-utilizing enzymes on the Bacterial tree [87]. An analysis of the *Cyanobacteria*, using Bayesian molecular clocks, calibrated with microfossils, suggest that *sodA* did not appear in this phylum until after the GOE [88]. Thus, the existence of *sodA* in the *Nitrospirota* LCA gene cluster set suggests this phylum originated after there was appreciable oxygen on Earth [87]. Gene clusters annotated as terminal oxidases are sporadically present in the *Thermodesulfovibrio* (Supplementary Figure 10). None of these gene clusters passed the thresholds for the enriched or LCA datasets used in this analysis, but gene reconciliation methods place the origination of one of these gene clusters at the root of the *Nitrospirota* tree (GC_00005908 – cbb-3 type cytochrome oxidase) (Supplementary Figure 10). Recently, the co-occurrence of the WLP and facultative aerobic respiration in *Thermodesulfovibrio* assemblies sampled from the terrestrial subsurface has been reported [38]. It appears the co-occurrence of these metabolic strategies in this phylum could be a widespread trait inherited from an ancestor which evolved during the period of oxygen accumulation on Earth [87].

### Metabolic expansion and progression

These analyses demonstrate that there is a metabolic progression throughout the history of *Nitrospirota* that is partially replicated in *Nitrospinota* (Fig. 6). The *Thermodesulfovibrio* and *UBA9217* are primarily sulfate reducers that utilize C1 compounds and hydrogen as electron sources, although other metabolisms such as sulfur disproportionation, sulfur oxidation, and nitrate reduction are documented (Figs. 4–6) [35, 37–40]. After these groups is the class *RBG 16-64-22* and *Nitrospiria* order *HDB-SIOI813*, which contain the *dsr* genes for rDSR and other sulfur-intermediate metabolisms [61, 71] (Fig. 6). Next is the *Nitrospirota* order *SBBL01*, containing recently described manganese-oxidizers [77, 78]. Genomes in this clade contain unique cytochromes involved in manganese oxidation that have been discussed in detail (Fig. 8, Supplementary Figure 10) [78].

The *nxr* genes responsible for nitrite-based metabolisms, originated early in the order *Nitrospirales* and were likely transferred from the *Planctomycetota* (Figs. 3, 6–8) [19]. The genes responsible for comammox (*pmo-amoABC* K10944-46, *hoa* K10535) originated at the base of the family *Nitrospiraceae* and are most closely related to order *Nitrosomonadales* (*Nitrosomonadacea* in GTDB) according to the taxonomy of the best eggNOG seed ortholog, which has been previously reported [26, 30]. These observations suggest *Nitrospirota* gained its comammox abilities by multiple gene acquisitions from different bacterial phyla. These patterns show *Nitrospirota* progressing from sulfate reduction to other sulfur-compound metabolisms, and then manganese oxidation, nitrite oxidation, and comammox (Figs. 6, 8).

The *Nitrospinota* show a partial replication of the metabolic progression of the *Nitrospirota* (Fig. 8C). The basal *Nitrospinota* classes *UBA7883* and *UBA9442* likely use rDSR and sulfur intermediates as an electron source, the same as *Nitrospirota* orders *RBG-16-64-22* and *HDB-SIOI813* (Figs. 4, 8) [61]. The phylogeny of the *dsrA* gene cluster shows that *Nitrospinota dsrA* sequences (and *Nitrospirota* class *RBG-16-64-22*) share a common evolutionary origin that is different to the reductive *dsrA* sequences in *Thermodesulfovibrio* (Supplementary Figure 10). In addition, a gene cluster involved in sulfite oxidation (*soeA*) is present in the LCA of *Nitrospinota*, and the *soeB* subunit is enriched in the basal classes, indicating the metabolisms of sulfur-cycle intermediates by basal *Nitrospinota* (Figs. 6, 8 Supplemental Data Files 6, 8) [71–73]. The basal classes of *Nitrospinota* and intermediate branching groups of *Nitrospirota* encode genes for the use of oxygen as a terminal electron acceptor (Fig. 4, Supplementary Figure 10). This is parsimonious with the interpretation that *Nitrospinota* originated in a more oxygenated environment than the basal groups of *Nitrospirota*. These shared sulfur oxidation metabolisms among basal *Nitrospinota* groups and intermediate-branching *Nitrospirota* groups might suggest a similar time of origin. This is supported by the *dsrA* phylogeny, which shows oxidative *Nitrospirota* and *Nitrospinota dsrA* sequences originating from the same common ancestor (Supplementary Figure 10) [61].

Following these sulfur-cycling metabolisms, both phyla acquire the *nxrAB* genes needed for nitrite oxidation (Figs. 6–8). The phylogenetic analysis of the *nxrA* gene cluster GC_00000823 with GenBank *nxrA* demonstrates that *Nitrospinota nxrA* sequences are also closely related to *nxrA* sequence in *Planctomycetota* (Fig. 7) [19]. Interestingly, the *Nitrospirota* sequences forms a bootstrap-supported monophyletic clade branching next to the Planctomycetota, while the *Nitrospinota* sequences form a second bootstrap supported monophyletic clade branching after the initial split between *Nitrospirota*/*Planctomycetota* (Fig. 7). This suggests that the *nxr* genes in *Nitrospirota* and *Nitrospinota* were transferred from *Planctomycetota* at two different times. These independent acquisitions of *nxrA* suggests the order *Nitrospirales* existed prior to the transfer of *nxrA* to order *Nitrospinales*. This scenario is similar to multiple independent gene acquisitions that define the lineage specific metabolisms of *Thermoplasmatota* order *Lutacidiplasmatales* [89]. The vertical relationship of oxidative *dsrA* sequences, the independent horizontal acquisitions of *nxrA* and similar gene loss/gain patterns depict an entangled and partially replicated evolution in the staggered history of these sister phyla.

## Conclusions

Here we demonstrate that the ancestral metabolisms of early branching clades for the sister phyla *Nitrospirota* and *Nitrospinota* are markedly different than the later branching groups that have received much attention due to their ecological prominence, especially in the marine environment, and unique nitrogen-based metabolisms. Despite some differences in particular metabolic functions, the similar evolutionary histories of *Nitrospirota* and *Nitrospinota* demonstrate how multiple modes of evolution can shape closely related phyla that occupy similar ecological niches. These data demonstrate that gene loss, de novo origination, or lateral acquisition of new genes is a replicated pattern in later-branching clades of phyla whose extant subsurface-inhabiting members resemble ancestral lineages that initially evolved in a primordial habitat.

## Supplementary information

Supplmental Methods and Figures

Supplemental Data 1

Supplemental Data 2

Supplemental Data 3

Supplemental Data 4

Supplemental Data 5

Supplemental Data 6

Supplemental Data 7

## Data Availability

Supplemental Data file 1 and Supplemental Data file 2 contain the NCBI and IMG Assembly IDs and NCBI BioSample accessions for all assemblies used in this study. Newly generated SAG data used in this study are available under NCBI BioProject IDs PRJNA825747 (Atlantis Massif), PRJNA779602 (Lost City Hydrothermal Field), PRJNA853307 (BLM1 Inyo-1), and PRJNA842252 (Juan De Fuca). All data processing scripts used to perform this analysis are available here: https://github.com/ts-dangelo/bioinformatic_scripts_python.
