# Supplementary material for: Replicated life-history patterns and subsurface origins of the bacterial sister phyla Nitrospirota and Nitrospinota
Source: ISME J. 2023 Apr 3;17(6):891–902. doi: 10.1038/s41396-023-01397-x (PMC10203281; doi:10.1038/s41396-023-01397-x)
Supplement: Supplementary file 1 — Supplmental Methods and Figures [file 41396_2023_1397_MOESM1_ESM.pdf]

Supplemental Methods and Materials for

Replicated Life-History Patterns and Subsurface Origins of the Bacterial Sister Phyla *Nitrospirota* and *Nitrospinota*

Authors: Timothy D'Angelo[1], Jacqueline Goordial[2], Melody R. Lindsay[1], Julia McGonigle[1,3], Anne Booker[1], Duane Moser[4], Ramunas Stepanauskus[1] and Beth N. Orcutt[1].

Author Affiliations:

1: Bigelow Laboratory for Ocean Sciences, 60 Bigelow Drive, East Boothbay Maine, USA 04544

2: University of Guelph, School of Environmental Sciences, 50 Stone Road East Guelph, Ontario, N1G 2W1 Canada

3: Basepaws Pet Genetics, 1820 W. Carson Street, Suite 202-351 Torrance, CA 90501

4: Desert Research Institute, 755 East Flamingo Road, Las Vegas, Nevada, USA 89119

Corresponding Author: Beth N. Orcutt, borcutt@bigelow.org

Competing Interest Statement

The authors declare no competing financial interests

Creation of Single-Cell Amplified Genomes (SAGs) from new subsurface sample collections

Lost City Hydrothermal Vent Field hydrothermal fluids

Lost City hydrothermal fluid was collected from the Calypso vent using the ROV *Jason* dive J2-1108 in September 2018 during the AT42-01 expedition aboard the R/V *Atlantis*. Fluid sampling is described in detail elsewhere [6]. Briefly, fluid from venting chimneys was collected into sterile chambers and incubated with a 600 µM solution of ^{13}C-formate and 2-3 mL of $^{15}N_2$ gas for the duration of the dives which lasted ~14-24 hours. Once shipboard, subsamples were collected from sample IDs LC00521 J2.1108.17Sept.2009.HOGBIO2 and J.1108.17Sept.1853.HOGCHEM 16 SCG1 and preserved for flow cytometric sorting of cells based on SYTO-9 (plate AH-679) or RedoxSensor™ Green fluorescence (plate AH-958), respectively, as previously described [1]. SAG generation was done through Bigelow's Single Cell Genomic Center SAG Generation pipeline (S-201). Select wells were picked for deeper sequencing through the PostLoCoS (S-203) pipeline. These SAGs were deposited to NCBI under the BioProject ID PRJNA779602 and are listed in Supplemental Table 1.

Supplemental Table 1: New Assemblies under NCBI BioProject PRJNA779602, sampled from the Lost City Hydrothermal Field. Assembly name is used in Supplemental Data File 1 for further metadata on these assemblies. All assemblies were a part of the main dataset analyzed in the paper

BioSample	Accession	Assembly Name	GTDB Taxonomic Classification
SAMN27740587	JALPWH000000000	AH_679_I05	d__Bacteria;p__Nitrospirota;c__Thermodesulfovibrionia;o__Thermodesulfovibrionales;f__UBA9935
SAMN27740595	JALPWP000000000	AH_679_J10	d__Bacteria;p__Nitrospirota;c__Thermodesulfovibrionia;o__Thermodesulfovibrionales;f__UBA9935
SAMN27740604	JALPWY000000000	AH_679_O07	d__Bacteria;p__Nitrospinota;c__Nitrospinia;o__Nitrospinales;f__Nitrospinaceae;g__SCGCAAA288-L16
SAMN27740611	JALPXF000000000	AH_679_P17	d__Bacteria;p__Nitrospirota;c__Thermodesulfovibrionia;o__Thermodesulfovibrionales;f__UBA9935
SAMN27740613	JALPXH000000000	AH_958_A08	d__Bacteria;p__Nitrospirota;c__Thermodesulfovibrionia;o__Thermodesulfovibrionales;f__UBA9935;g__GWB2-47-37
SAMN27740620	JALPXO000000000	AH_958_D16	d__Bacteria;p__Nitrospirota;c__Thermodesulfovibrionia;o__Thermodesulfovibrionales;f__UBA9935
SAMN27740643	JALPYL000000000	AH_958_K20	d__Bacteria;p__Nitrospirota;c__Thermodesulfovibrionia;o__Thermodesulfovibrionales;f__UBA9935
SAMN27740644	JALPYM000000000	AH_958_L11	d__Bacteria;p__Nitrospirota;c__Thermodesulfovibrionia;o__Thermodesulfovibrionales;f__UBA9935
SAMN27740645	JALPYN000000000	AH_958_L16	d__Bacteria;p__Nitrospirota;c__Thermodesulfovibrionia;o__Thermodesulfovibrionales;f__UBA9935

BLM1-Inyo 1 Terrestrial Subsurface

Terrestrial Subsurface Groundwater. Monitoring well Inyo-BLM 1 (36.4004N, -116.4692W, 694 masl.) was drilled to 871 m in 2007 by The Hydrodynamics Group, LLC. (Redmond, WA) for Inyo County, California and samples the discharge zone of the Death Valley Regional Flow System (DVRFS) of the Basin and Range (B&R) physiographic province of the western United States. The well is continuously cased in low-carbon steel to 750 mbls, bypassing shallower aquifers associated with lake sediments, volcanic tuff, and valley fill alluvium and produces hot (~60 ºC), anoxic groundwater from a faulted zone in Paleozoic sedimentary rocks that hosts the "Lower Carbonate Aquifer [LCA]") of the DVRFS [7]. The LCA is recharged via secondary fracture permeability and "interbasin flow" by groundwater derived from montane zones 10 to >100 km to the North and East, ultimately discharging to the Death Valley salt pan, at ~86 masl, the lowest point in North America [8,9]. Halford and Jackson (2020) posit that the DVRFS is compartmentalized into a shallow, high-transmissivity zone within ~500 m of the water table where nearly all flow occurs; and a deeper, low-transmissivity zone, which is the source of water to Inyo-BLM 1 [10]. To assure pristine samples, groundwater was obtained from a gas-tight manifold after 30 h of continuous high-volume pumping (200 gallon (757 L) per minute and processed immediately onsite in a nitrogen-flushed glovebag. Samples for flow cytometric sorting of cells based on SYTO-9 (plate AM-294) or RedoxSensor™ Green fluorescence (plate AM-297), were preserved on dry ice in the field and held at -80ºC prior to SAG generation through Bigelow Lab's Single Cell Genomic Center SAG Generation pipeline (S-211).

Supplemental Table 2: New SAG Assemblies from Inyo-BLM1 under NCBI BioProject PRJNA853307. Assembly name column is used in Supplemental File 1 for further metadata on these assemblies.

BioSample	Accession	Assembly Name	GTDB Taxonomic Classification
SAMN29377803	SAMN29377803	AM_294_N06	d__Bacteria;p__Nitrospirota;c__Thermodesulfovibrionia;o__Thermodesulfovibrionales;f__SM23-35;g__JACAFF01
SAMN29377717	SAMN29377717	AM_297_G21	d__Bacteria;p__Nitrospirota;c__Thermodesulfovibrionia;o__Thermodesulfovibrionales;f__SM23-35;g__JACAFF01

Resequencing of SAGs from Atlantis Massif subsurface rock samples

Prior work describes the collection and original genomic sequencing and assembly of SAGs from rock samples collected from the subsurface of the Atlantis Massif, an underwater mountain that hosts the Lost Coty Hydrothermal Vent field [1]. Here, these SAGs were re-sequenced to produce more complete assemblies using the S-203 workflow described by the Single Cell Genomics Center (SCGC) at the Bigelow Laboratory for Ocean Sciences (https://scgc.bigelow.org/capabilities/service-description/), as published elsewhere [2]. These SAGs were assigned taxonomy using the GTDB-tk classifier (version r202) and a pairwise Average Nucleotide Identity (ANI) was performed on all SAGs [3, 4]. In order to produce a more complete assembly representing the underlying population of cells, the reads that produced SAGs that received the same taxonomic assignment by GTDB-tk and had >98% ANI were co-assembled using spades (using the –sc flag) [5]. These SAGs were deposited to NCBI under the BioProject ID PRJNA825747 and are listed in Supplemental Table 3.

Supplemental Table 3: New SAG Assemblies from Atlantis Massif under NCBI BioProject PRJNA825747. Assembly name column is used in Supplemental File 1 for further metadata on these assemblies. Bolded Assemblies were used in the main analysis in the paper. Each Letter-Number code after AH_259 represents a single cell. Co-assemblies contain multiple codes equal to the number cells included in assembly (two or three).

BioSample	Accession	Assembly Name	GTDB Taxonomic Classification
**SAMN27544102**	**JALLOE000000000**	**AH_259_D15_M11_P09**	**d__Bacteria;p__Nitrospirota;c__Nitrospiria;o__Nitrospirales;f__Nitrospiraceae**
SAMN27544101	JALLOF000000000	AH_259_B05_G02_I21	d__Bacteria;p__Nitrospinota_A;c__UBA8248
SAMN27544098	JALLOI000000000	AH-259-F20	d__Bacteria;p__Nitrospinota_A;c__UBA8248
SAMN27544096	JALLOK000000000	AH-259-D05	d__Bacteria;p__Acidobacteriota;c__UBA6911;o__RPQK01;f__RPQK01;g__;s__

Detailed Description of Phylogenomic Multiple Sequence Alignment (MSA) construction.

Diamond was used to search the assemblies for single copy marker genes from the Bac120 database using the following parameters: blastp --quiet --threads 1 --outfmt 6 --more-sensitive --id 50 --max-hsps 35 -k 0 [3, 14]. MAFFT was used to align the protein sequences for each marker gene using the –auto flag [15]. The MSAs were trimmed using trimAL with the –gappyout option [16]. Additionally, columns with more than 85% gaps or with >95% identical amino acid composition were removed. Sequences with >75% gaps were also removed. Trident was used to score the columns to identify the most phylogenetically informative positions [17]. The top 25 were used from each MSA. The resulting data from each MSA was then concatenated into the final alignment used for phylogenomic tree inference. This alignment and trimming procedure resulting in 1900 positions for the outgroup-rooted Nitrospirota alignment (Figure 1, 5, 6) and 3958 for the MAD rooted alignment without the use of an outgroup (Figures 3, 8). The alignment for outgroup-rooted Nitrospinota contained 2425 positions and the M.A.D tree alignment contained 2274 positions.

Supplemental References:

1. Goordial J, D'angelo T, Labonté JM, Poulton NJ, Brown JM, Stepanauskas R, *et al*. Microbial diversity and function in shallow subsurface sediment and oceanic lithosphere of the Atlantis Massif. mBio. 2021 Aug 31;12(4):e00490-21. https://doi.org/10.1128/mbio.00490-21.
2. Stepanauskas R, Fergusson EA, Brown J, Poulton NJ, Tupper B, Labonté JM, *et al*. Improved genome recovery and integrated cell-size analyses of individual uncultured microbial cells and viral particles. Nature Communications. 2017 Jul 20;8(1):84. https://doi.org/10.1038/s41467-017-00128-z.
3. Parks DH, Chuvochina M, Waite DW, Rinke C, Skarshewski A, Chaumeil PA, *et al*. A standardized bacterial taxonomy based on genome phylogeny substantially revises the tree of life. Nature Biotechnology. 2018 Nov;36(10):996-1004. https://doi.org/10.1038/nbt.4229.
4. Jain C, Rodriguez-R LM, Phillippy AM, Konstantinidis KT, Aluru S. High throughput ANI analysis of 90K prokaryotic genomes reveals clear species boundaries. Nature Communications. 2018 Nov 30;9(1):5114. https://doi.org/10.1038/s41467-018-07641-9.
5. Bankevich A, Nurk S, Antipov D, Gurevich AA, Dvorkin M, Kulikov AS, *et al*. SPAdes: a new genome assembly algorithm and its applications to single-cell sequencing. Journal of Computational Biology. 2012 May 1;19(5):455-77. https://doi.org/10.1089/cmb.2012.0021.
6. Brazelton WJ, McGonigle JM, Motamedi S, Pendleton HL, Twing KI, Miller BC, *et al*. Metabolic strategies shared by basement residents of the Lost City hydrothermal field.

Applied and Environmental Microbiology. 2022 Sep 13;88(17):e00929-22. https://doi.org/10.1128/aem.00929-22.

7. Bredehoeft J, King M. Potential contaminant transport in the regional Carbonate Aquifer beneath Yucca Mountain, Nevada, USA. Hydrogeology Journal. 2010;3(18):775-89. 10.1007/s10040-009-0550-z.
8. Winograd IJ. Interbasin groundwater flow in south central Nevada: A further comment on the discussion between Davisson et. al..[1999a, 1999b] and Thomas [1999]. Water Resources Research. 2001 Feb;37(2):431-3. https://doi.org/10.1029/2000WR900345.
9. Belcher WR, Bedinger MS, Back JT, Sweetkind DS. Interbasin flow in the Great Basin with special reference to the southern Funeral Mountains and the source of Furnace Creek springs, Death Valley, California, US. Journal of Hydrology. 2009 May 5;369(1-2):30-43. https://doi.org/10.1016/j.jhydrol.2009.02.048.
10. Halford KJ, Jackson TR. Groundwater characterization and effects of pumping in the Death Valley regional groundwater flow system, Nevada and California, with special reference to Devils Hole. US Geological Survey; 2020. https://doi.org/10.3133/pp1863.
11. Orcutt BN, Bach W, Becker K, Fisher AT, Hentscher M, Toner BM, *et al*. Colonization of subsurface microbial observatories deployed in young ocean crust. The ISME Journal. 2011 Apr;5(4):692-703. https://doi.org/10.1038/ismej.2010.157.
12. Carr SA, Jungbluth SP, Eloe-Fadrosh EA, Stepanauskas R, Woyke T, Rappé MS, *et al*. Carboxydotrophy potential of uncultivated Hydrothermarchaeota from the subseafloor crustal biosphere. The ISME Journal. 2019 Jun;13(6):1457-68. https://doi.org/10.1038/s41396-019-0352-9.

13. Jungbluth SP, Amend JP, Rappé MS. Metagenome sequencing and 98 microbial genomes from Juan de Fuca Ridge flank subsurface fluids. Scientific Data. 2017 Mar 28;4(1):1-1. https://doi.org/10.1038/sdata.2017.37

14. Buchfink B, Xie C, Huson DH. Fast and sensitive protein alignment using DIAMOND. Nature Methods. 2015 Jan;12(1):59-60. https://doi.org/10.1038/nmeth.3176.

15. Katoh K, Standley DM. MAFFT multiple sequence alignment software version 7: improvements in performance and usability. Molecular Biology and Evolution. 2013 Jan 16;30(4):772-80. https://doi.org/10.1093/molbev/mst010.

16. Capella-Gutiérrez S, Silla-Martínez JM, Gabaldón T. trimAl: a tool for automated alignment trimming in large-scale phylogenetic analyses. Bioinformatics. 2009 Aug 1;25(15):1972-3. https://doi.org/10.1093/bioinformatics/btp348.

17. Valdar WS. Scoring residue conservation. Proteins: Structure, Function, and Bioinformatics. 2002 Aug 1;48(2):227-41. https://doi.org/10.1002/prot.10146.

Supplemental Data File Descriptions:

Supplemental Data 1: Assembly names, CheckM assembly statistics and GTDB classifications for all assemblies used in the analysis, including sampling habitat used in ASR analysis and coordinates of sampling site.

Supplemental Data 2: Consensus annotations for gene clusters enriched in the basal *Nitrospirota* classes as delineated by the hierarchical clustering displayed in Supplemental Figure 5. Annotation data from KOFAMSCAN and eggNOG are summarized

Supplemental Data 3: Consensus annotations for gene clusters enriched in the *Nitrospirota* class *Nitrospiria* as delineated by the hierarchical clustering displayed in Supplemental Figure 5. Annotation data from KOFAMSCAN and eggNOG are summarized

Supplemental Data 4: Consensus annotations for gene clusters identified to have >0.5 posterior probability of being present in the Last Common Ancestor of *Nitrospirota*. Annotation data from KOFAMSCAN and eggNOG are summarized

Supplemental Data 5: Consensus annotations for gene clusters enriched in the basal *Nitrospinota* classes as delineated by the hierarchical clustering displayed in Supplemental Figure 5. Annotation data from KOFAMSCAN and eggNOG are summarized

Supplemental Data 6: Consensus annotations for gene clusters enriched in the *Nitrospinota* class *Nitrospinia* as delineated by the hierarchical clustering displayed in Supplemental Figure 5. Annotation data from KOFAMSCAN and eggNOG are summarized

Supplemental Data 7: Consensus annotations for gene clusters identified to have >0.5 posterior probability of being present in the Last Common Ancestor of *Nitrospinota*. Annotation data from KOFAMSCAN and eggNOG are summarized

Supplemental Figures

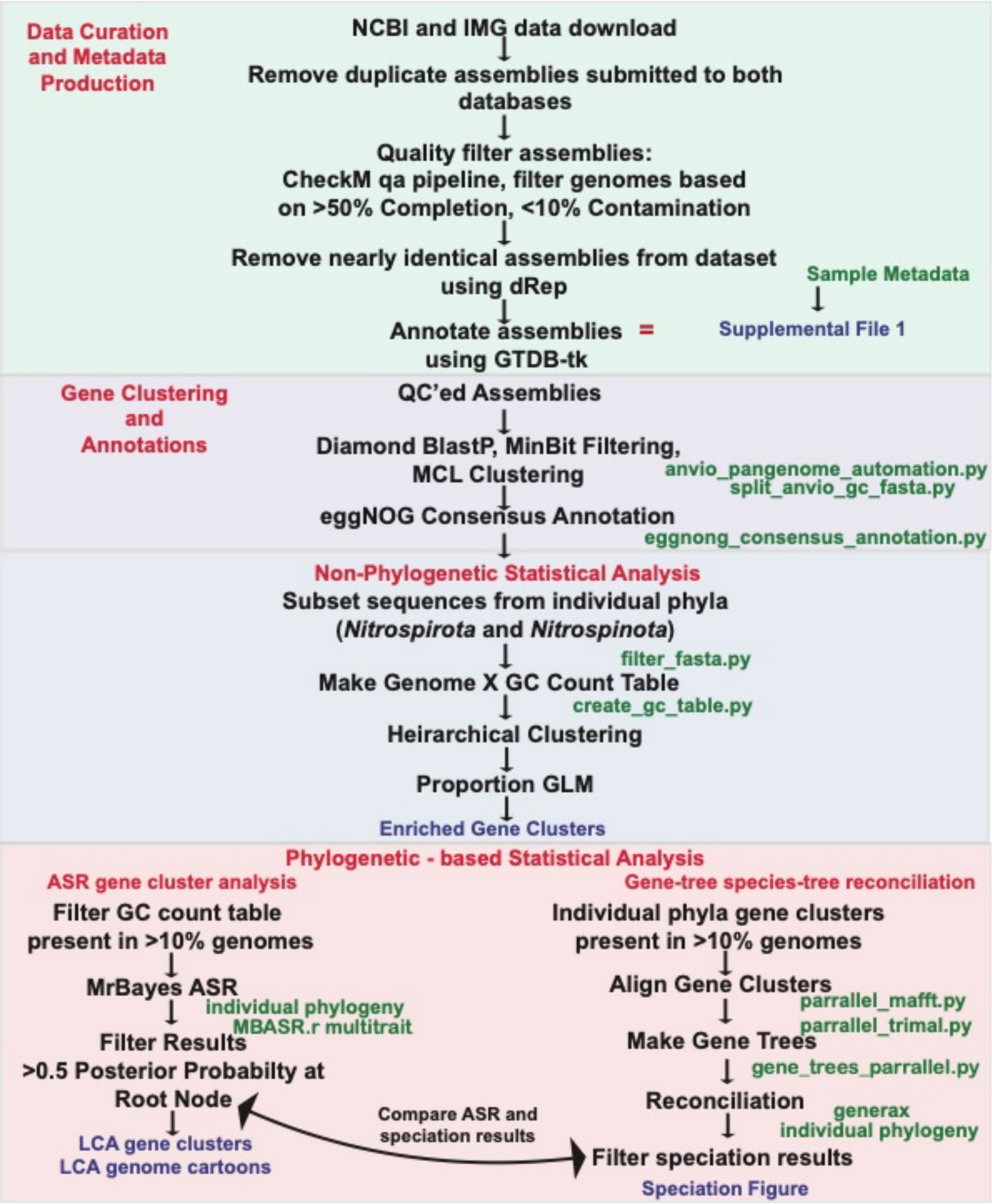

Supplemental Figure 1: Workflow diagram for the bioinformatic analyses performed for this study. The resulting set of assemblies with metadata described in the top box is Supplemental File 1. All scripts used to process the data can be found at: https://github.com/ts-dangelo/bioinformatic_scripts_python

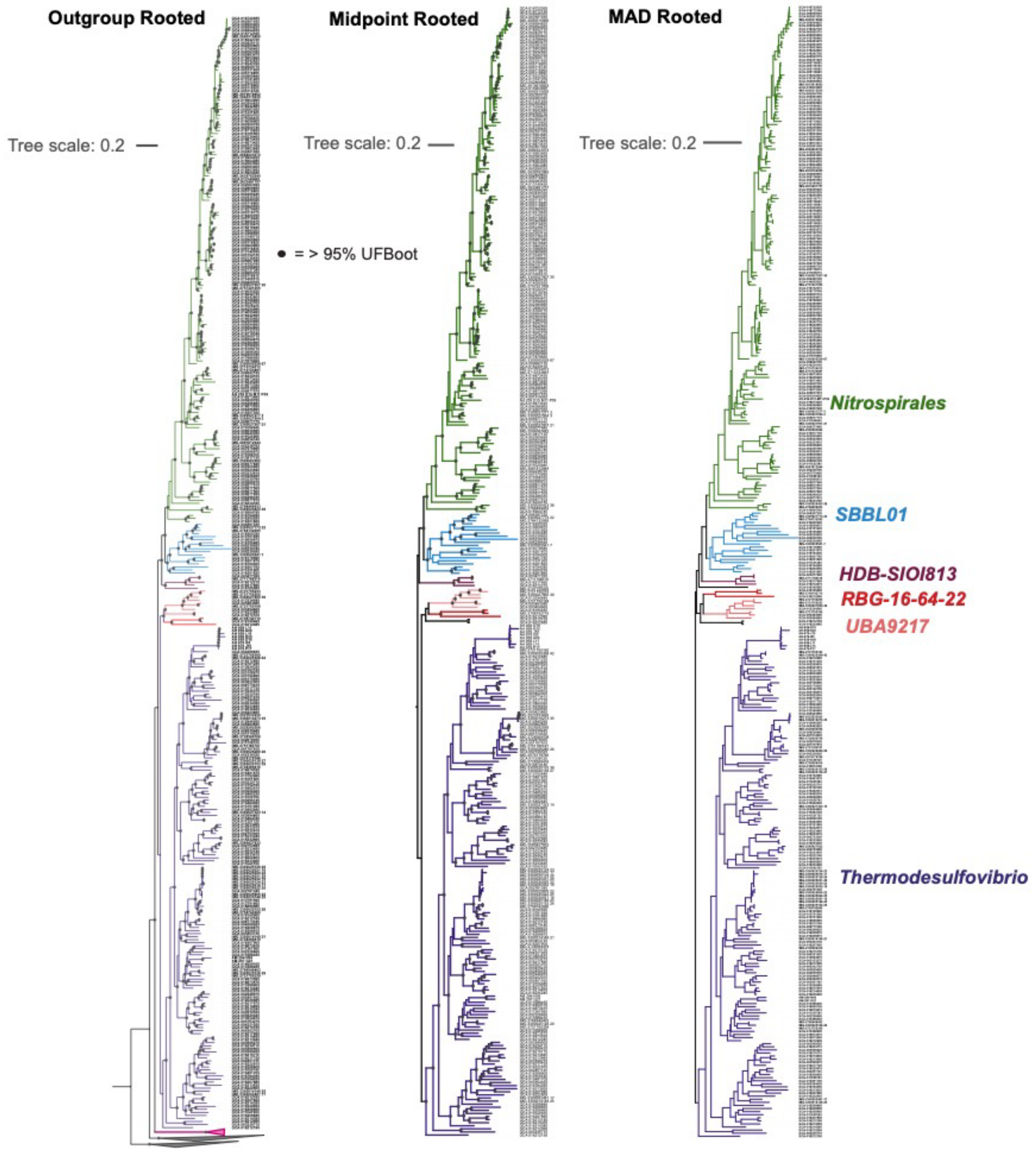

Supplemental Figure 2: Phylogenomic comparisons of tree rooting methods for *Nitrospirota* All phylogenomic trees are the consensus tree produced used the Bac120 marker set (min 12 genes) using the PhyloPhlan pipeline to create the alignments and IqTree with ModelFinder and 1000 ultra-fast non-parametric bootstraps. The model LG+F+G4 was chosen via the Bayesian Information Criterion (BIC) score by Model Finder. The outgroup rooted tree is rooted in the branch separating the *Desulfobacterota_D* from *Desulfobacterota* (class

Thermodesulfobacteria), and includes *Nitrospirota_A* (collapsed pink clade). The midpoint rooted tree is the consensus tree of 1000 ultra-fast non-parametric bootstrap trees. The MAD tree is the same tree as the midpoint tree but rooted using Minimal Ancestor Deviation. The outgroup rooted tree is used for Figures 1, 5, 6 and the MAD rooted tree was used for LCA and reconciliation-based methods (Fig. 3, 8).

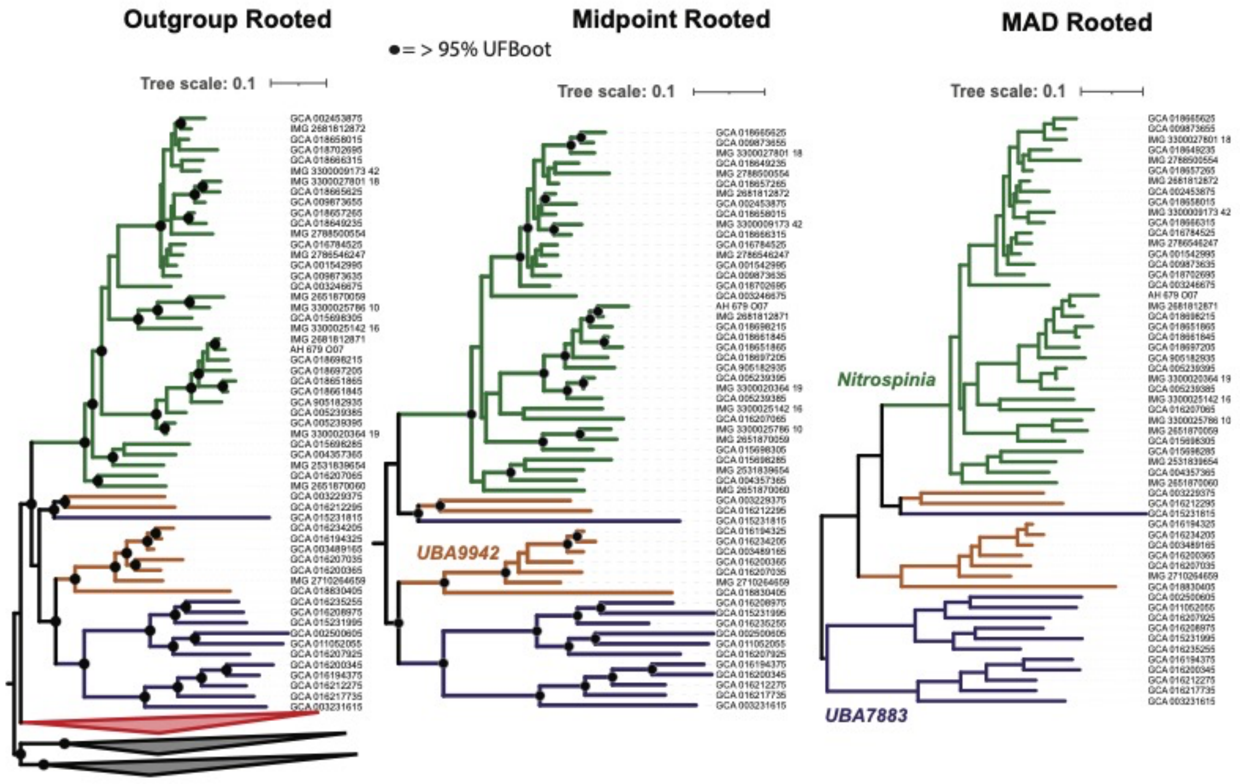

Supplemental Figure 3: Phylogenomic comparisons of tree rooting methods for *Nitrospinota*. All phylogenomic trees are the consensus tree produced used the Bac120 marker set (min 12 genes) using the PhyloPhlan pipeline to create the alignments and IQ-TREE with ModelFinder and 1000 ultra-fast non-parametric bootstraps. The model LG+F+G4 was chosen via the Bayesian Information Criterion (BIC) score by Model Finder. The outgroup rooted tree is rooted in the branch separating the *Desulfobacterota_D* from *Desulfobacterota* (class *Thermodesulfobacteria*), and includes *Nitrospinota_A* (collapsed pink clade). The midpoint rooted tree is the consensus tree of 1000 ultra-fast non-parametric bootstrap trees. The MAD tree is the same tree as the midpoint tree but rooted using Minimal Ancestor Deviation. The outgroup rooted tree is used for Figures 1, 5, 6 and the MAD rooted tree was used for LCA and reconciliation-based methods (Figures 3, 6).

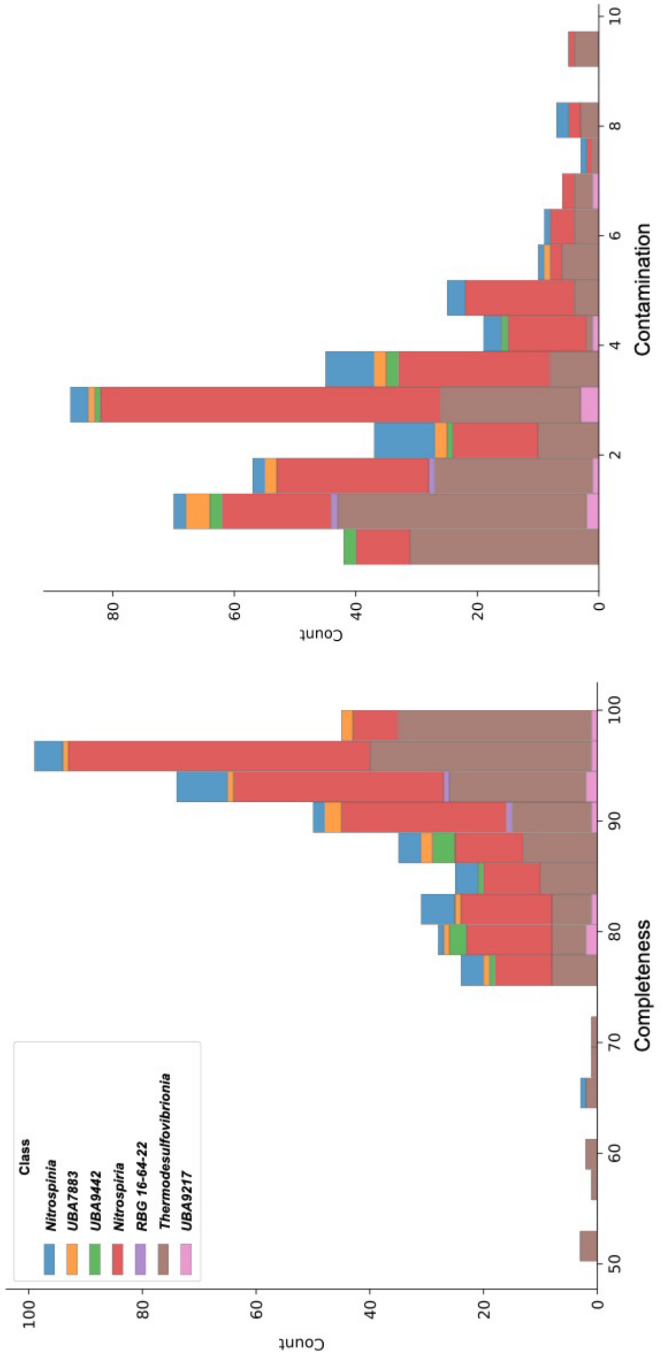

Supplemental Figure 4: CheckM Completion and Contamination histograms for *Nitrospirota* and *Nitrospinota* assemblies with detailed metadata in Supplemental File 1 colored by *class* taxonomic rank.

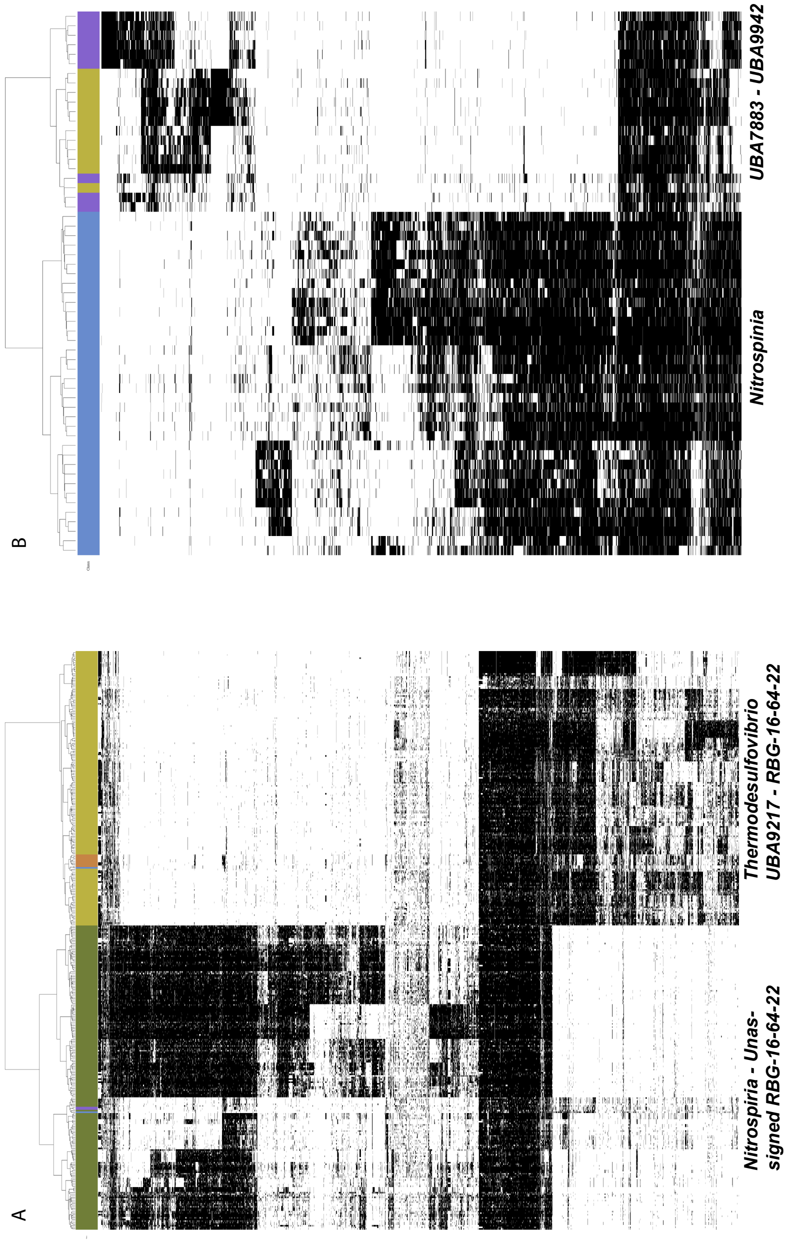

Supplemental Figure 5: Heatmaps of binary hierarchical clustering using Ward's Linkage Method for *Nitrospirota* (A) and *Nitrospinota* (B). These groupings were used for downstream statistical analyses.

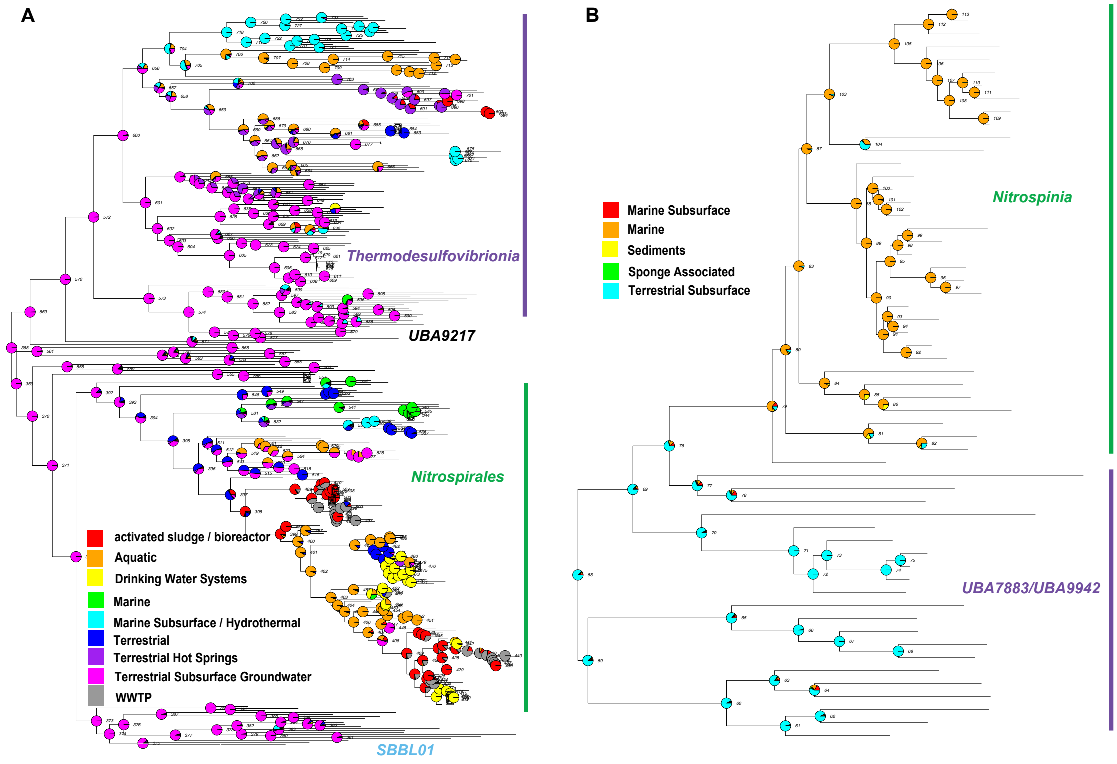

Supplemental Figure 6: Ancestral State Reconstruction (ASR) of possible habitat of the ancestor of *Nitrospirota* (A) and *Nitrospinota* (B). The habitat sampled for the assemblies were coded as variables in MrBayes ASR. The pie chart at each internal node of the tree represents the posterior probability of the given node existing in a particular habitat type. Metadata for these analyses are available in Supplemental File 1.

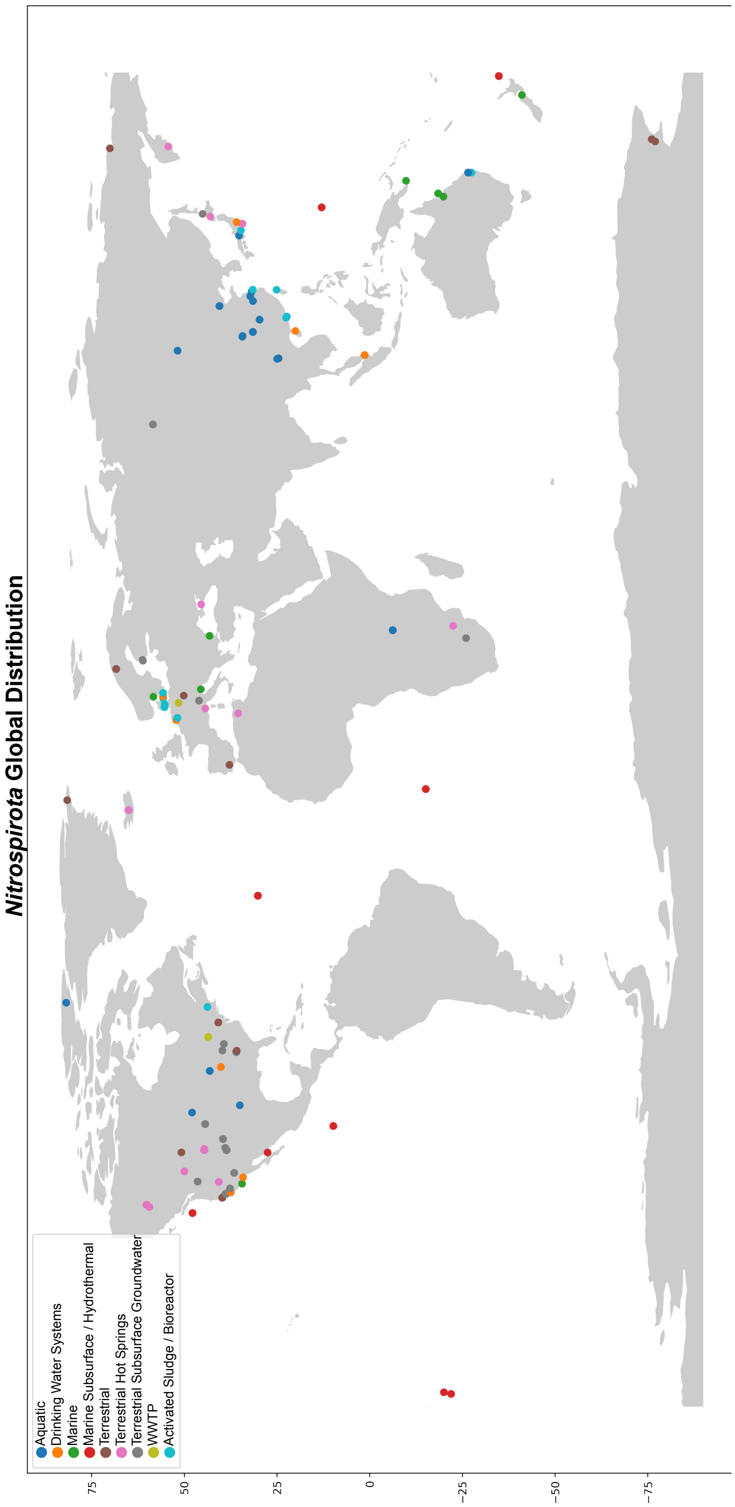

Supplemental Figure 7: Map of Global *Nitrospirota* distribution. Metadata for these points are available in Supplemental File 1.

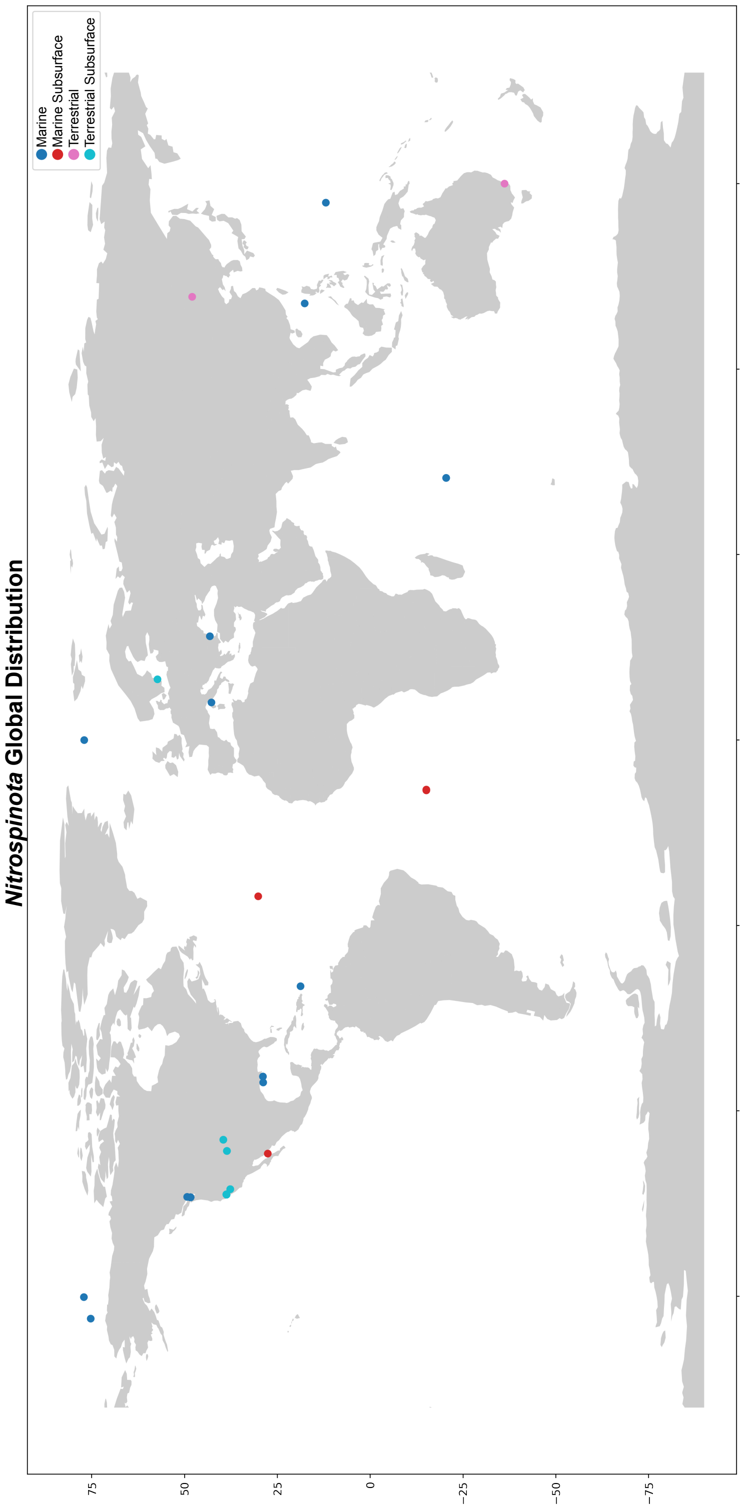

Supplemental Figure 8: Map of Global *Nitrospinota* distribution. Metadata for these points are available in Supplemental File 1.

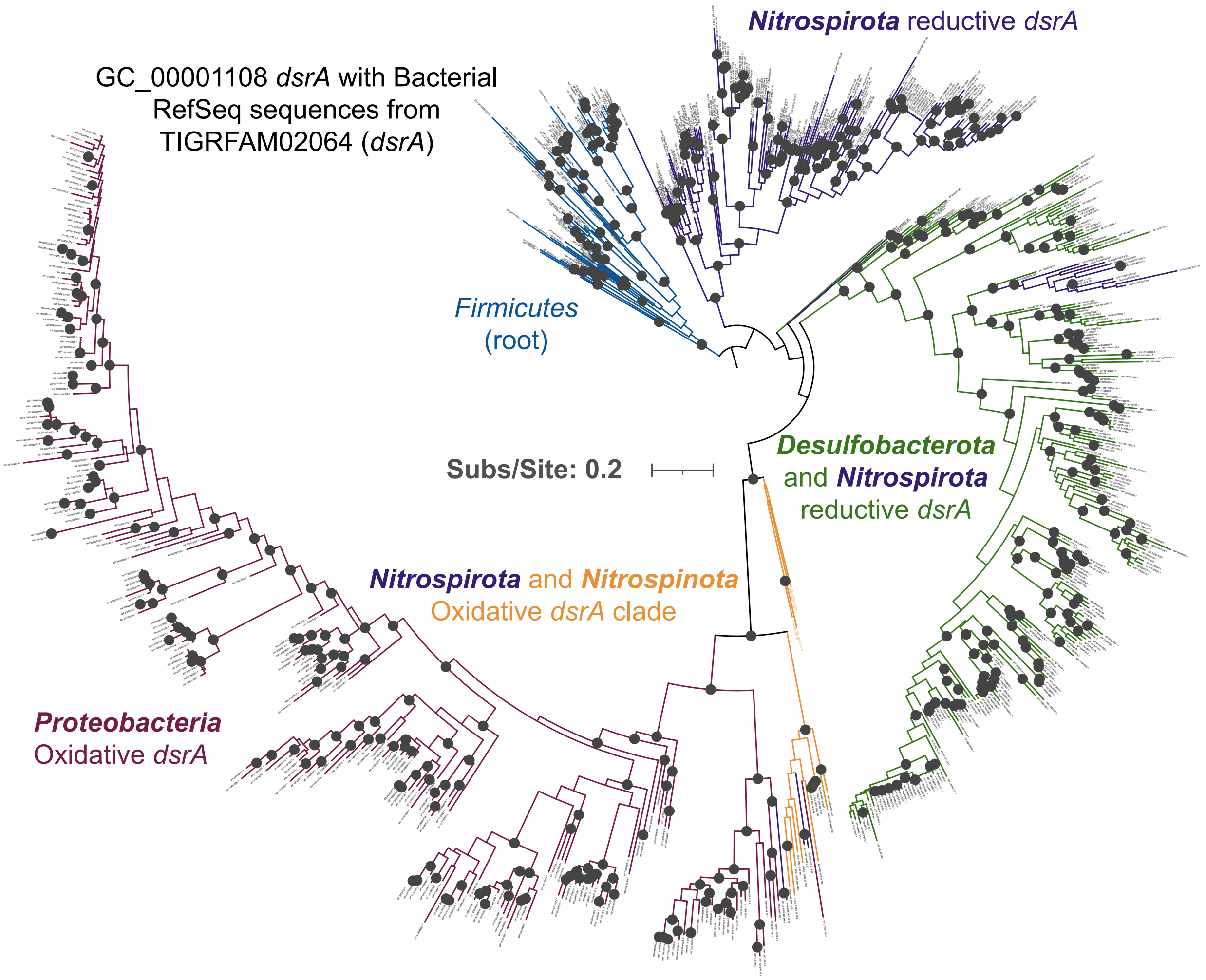

Supplementary Figure 9: A phylogeny for *dsrA* gene cluster GC_00001108 with RefSeq-quality Bacterial sequences in TIGRFAM02064. The reductive *Nitrospirota dsrA* sequences form a monophyletic clade, with one instance of a clade of *Nitrospirota* sequences existing within *Desulfobacterota*. Presumed oxidative rDSR *dsrA* sequences from both *Nitrospirota* and *Nitrospinota* branch closely to sulfide-oxidizing *Proteobacteria*.

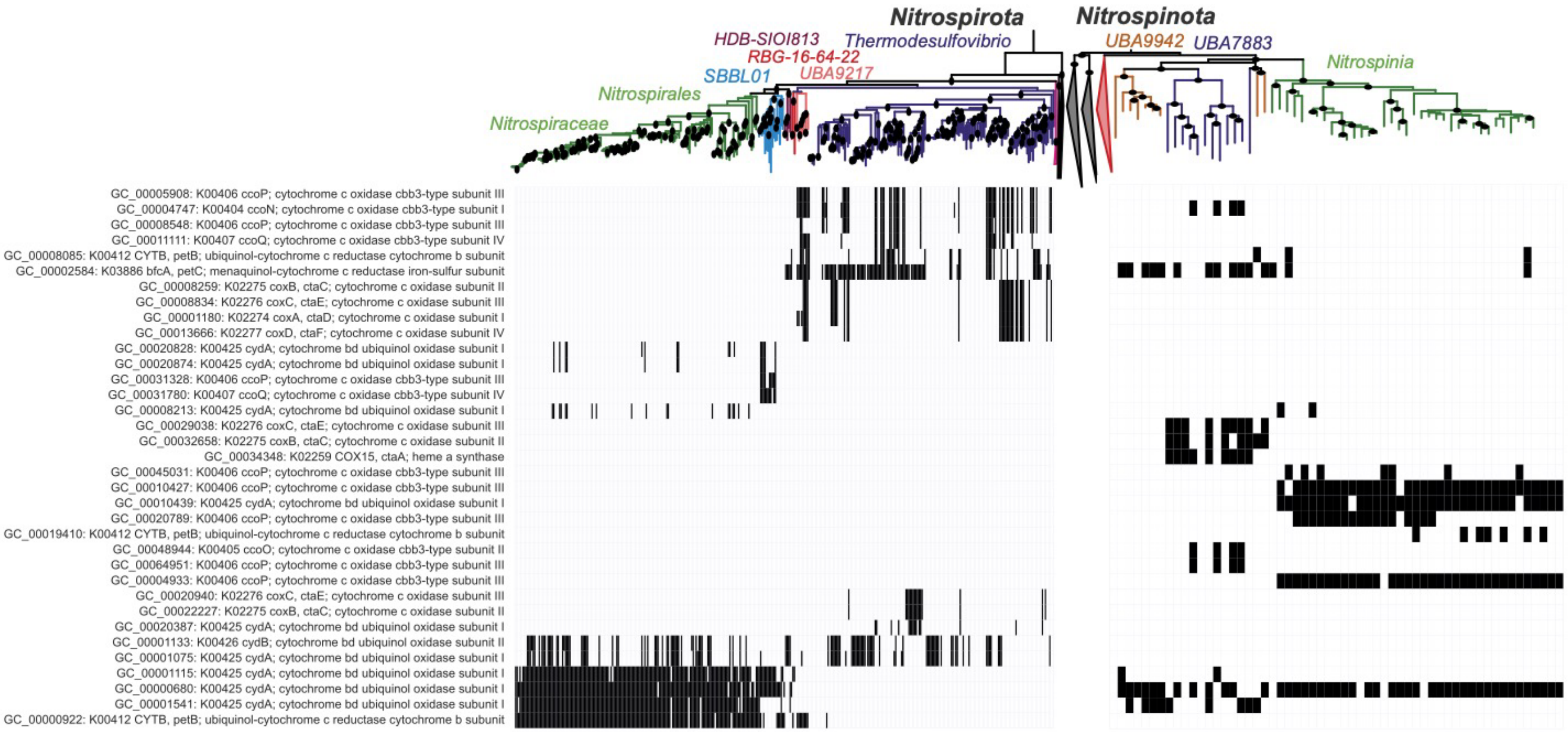

Nitrospirota
Nitrospinota
HDB-SIOI813
RBG-16-64-22
Thermodesulfovibrio
UBA9942
UBA7883
Nitrospinia
SBBL01
UBA9217
Nitrospirales
Nitrospiraceae
GC_00005908: K00406 ccoP; cytochrome c oxidase cbb3-type subunit III
GC_00004747: K00404 ccoN; cytochrome c oxidase cbb3-type subunit I
GC_00008548: K00406 ccoP; cytochrome c oxidase cbb3-type subunit III
GC_00011111: K00407 ccoQ; cytochrome c oxidase cbb3-type subunit IV
GC_00008085: K00412 CYTB, petB; ubiquinol-cytochrome c reductase cytochrome b subunit
GC_00002584: K03886 bfcA, petC; menaquinol-cytochrome c reductase iron-sulfur subunit
GC_00008259: K02275 coxB, ctaC; cytochrome c oxidase subunit II
GC_00008834: K02276 coxC, ctaE; cytochrome c oxidase subunit III
GC_00001180: K02274 coxA, ctaD; cytochrome c oxidase subunit I
GC_00013666: K02277 coxD, ctaF; cytochrome c oxidase subunit IV
GC_00020828: K00425 cydA; cytochrome bd ubiquinol oxidase subunit I
GC_00020874: K00425 cydA; cytochrome bd ubiquinol oxidase subunit I
GC_00031328: K00406 ccoP; cytochrome c oxidase cbb3-type subunit III
GC_00031780: K00407 ccoQ; cytochrome c oxidase cbb3-type subunit IV
GC_00008213: K00425 cydA; cytochrome bd ubiquinol oxidase subunit I
GC_00029038: K02276 coxC, ctaE; cytochrome c oxidase subunit III
GC_00032658: K02275 coxB, ctaC; cytochrome c oxidase subunit II
GC_00034348: K02259 COX15, ctaA; heme a synthase
GC_00045031: K00406 ccoP; cytochrome c oxidase cbb3-type subunit III
GC_00010427: K00406 ccoP; cytochrome c oxidase cbb3-type subunit III
GC_00010439: K00425 cydA; cytochrome bd ubiquinol oxidase subunit I
GC_00020789: K00406 ccoP; cytochrome c oxidase cbb3-type subunit III
GC_00019410: K00412 CYTB, petB; ubiquinol-cytochrome c reductase cytochrome b subunit
GC_00048944: K00405 ccoO; cytochrome c oxidase cbb3-type subunit II
GC_00064951: K00406 ccoP; cytochrome c oxidase cbb3-type subunit III
GC_00004933: K00406 ccoP; cytochrome c oxidase cbb3-type subunit III
GC_00020940: K02276 coxC, ctaE; cytochrome c oxidase subunit III
GC_00022227: K02275 coxB, ctaC; cytochrome c oxidase subunit II
GC_00020387: K00425 cydA; cytochrome bd ubiquinol oxidase subunit I
GC_00001133: K00426 cydB; cytochrome bd ubiquinol oxidase subunit II
GC_00001075: K00425 cydA; cytochrome bd ubiquinol oxidase subunit I
GC_00001115: K00425 cydA; cytochrome bd ubiquinol oxidase subunit I
GC_00000680: K00425 cydA; cytochrome bd ubiquinol oxidase subunit I
GC_00001541: K00425 cydA; cytochrome bd ubiquinol oxidase subunit I
GC_00000922: K00412 CYTB, petB; ubiquinol-cytochrome c reductase cytochrome b subunit

Supplemental Figure 10: Presence/absence heatmap of gene clusters with consensus annotations for oxygen-reducing terminal oxidases and oxygen-utilizing electron transport components from KEGG map00190. The rows are ordered by hierarchical clustering of the present/absence patterns in *Nitrospirota*.
